# Enhancing AI Chatbot Responses in Health Care: The SMART Prompt Structure in Head and Neck Surgery

**DOI:** 10.1002/oto2.70075

**Published:** 2025-01-16

**Authors:** Luigi Angelo Vaira, Jerome R. Lechien, Vincenzo Abbate, Guido Gabriele, Andrea Frosolini, Andrea De Vito, Antonino Maniaci, Miguel Mayo‐Yáñez, Paolo Boscolo‐Rizzo, Alberto Maria Saibene, Fabio Maglitto, Giovanni Salzano, Gianluigi Califano, Stefania Troise, Carlos Miguel Chiesa‐Estomba, Giacomo De Riu

**Affiliations:** ^1^ Maxillofacial Surgery Operative Unit, Department of Medicine, Surgery and Pharmacy University of Sassari Sassari Italy; ^2^ Department of Surgery, Mons School of Medicine, UMONS Research Institute for Health Sciences and Technology University of Mons (UMons) Mons Belgium; ^3^ Department of Otolaryngology–Head and Neck Surgery Elsan Hospital Paris France; ^4^ Head and Neck Section, Department of Neurosciences, Reproductive and Odontostomatological Science Federico II University of Naples Naples Italy; ^5^ Maxillofacial Surgery Unit, Department of Medical Biotechnologies University of Siena Siena Italy; ^6^ Department of Medicine, Surgery and Pharmacy University of Sassari Sassari Italy; ^7^ Department of Medicine and Surgery University of Enna Kore Enna Italy; ^8^ Otorhinolaryngology, Head and Neck Surgery Department Complexo Hospitalario Universitario A Coruña (CHUAC) A Coruña Spain; ^9^ Section of Otolaryngology, Department of Medical, Surgical and Health Sciences University of Trieste Trieste Italy; ^10^ Otolaryngology Unit, Department of Health Sciences, Santi Paolo e Carlo Hospital University of Milan Milan Italy; ^11^ Maxillo‐Facial Surgery Unit University of Bari “Aldo Moro” Bari Italy; ^12^ Department of Neurosciences, Reproductive and Odontostomatological Science Federico II University of Naples Naples Italy; ^13^ Department of Otorhinolaryngology–Head and Neck Surgery Hospital Universitario Donostia San Sebastian Spain

**Keywords:** AI, artificial intelligence, ChatGPT, maxillofacial surgery, otorhinolaryngology, prompt engineering

## Abstract

**Objective:**

This study aims to evaluate the impact of prompt construction on the quality of artificial intelligence (AI) chatbot responses in the context of head and neck surgery.

**Study Design:**

Observational and evaluative study.

**Setting:**

An international collaboration involving 16 researchers from 11 European centers specializing in head and neck surgery.

**Methods:**

A total of 24 questions, divided into clinical scenarios, theoretical questions, and patient inquiries, were developed. These questions were entered into ChatGPT‐4o both with and without the use of a structured prompt format, known as SMART (Seeker, Mission, AI Role, Register, Targeted Question). The AI‐generated responses were evaluated by experienced head and neck surgeons using the Quality Analysis of Medical Artificial Intelligence instrument (QAMAI), which assesses accuracy, clarity, relevance, completeness, source quality, and usefulness.

**Results:**

The responses generated using the SMART prompt scored significantly higher across all QAMAI dimensions compared to those without contextualized prompts. Median QAMAI scores for SMART prompts were 27.5 (interquartile range [IQR] 25‐29) versus 24 (IQR 21.8‐25) for unstructured prompts (*P* < .001). Clinical scenarios and patient inquiries showed the most significant improvements, while theoretical questions also benefited, but to a lesser extent. The AI's source quality improved notably with the SMART prompt, particularly in theoretical questions.

**Conclusion:**

This study suggests that the structured SMART prompt format significantly enhances the quality of AI chatbot responses in head and neck surgery. This approach improves the accuracy, relevance, and completeness of AI‐generated information, underscoring the importance of well‐constructed prompts in clinical applications. Further research is warranted to explore the applicability of SMART prompts across different medical specialties and AI platforms.

In recent years, the integration of artificial intelligence (AI) within the health care sector has rapidly expanded, offering unprecedented opportunities to enhance patient care, streamline administrative processes, and support clinical decision‐making.^1^, ^2^, ^3^ Among the various AI applications, conversational agents, commonly known as chatbots, have gained significant attention for their potential to interact with users in a natural language format. These AI‐driven systems are being increasingly utilized for a wide range of purposes, including symptom checking, patient triage, mental health support, and health information dissemination.^4^, ^5^, ^6^, ^7^, ^8^, ^9^, ^10^

However, the effectiveness of AI chatbots in health care largely depends on the quality of the interactions they facilitate.^11^, ^12^ A key factor influencing these interactions is the way users formulate their prompts or queries.^13^, ^14^, ^15^ Unlike traditional search engines, where users might rely on keyword‐based queries, interactions with AI chatbots often involve more complex, context‐rich language, which can significantly affect the responses generated by these systems. This is particularly crucial in health care settings, where the accuracy, clarity, and relevance of information can have profound implications on patient outcomes. Providing adequate context to an AI chatbot is crucial for obtaining accurate and contextually appropriate responses. The quality of AI‐generated responses is significantly influenced by the amount and relevance of the information provided in the prompt.^16^ When a prompt lacks sufficient context, the AI is forced to make broader assumptions, which can lead to generalized or less accurate responses. Conversely, when a prompt includes detailed and specific information, such as patient history, symptoms, or the specific surgical context, the AI can tailor its responses more precisely to the needs of the user.

Despite the growing use of AI chatbots, there is a limited amount of research exploring how the structuring of prompts influences the quality of responses in health care‐related contexts.^13^, ^14^, ^15^ Understanding this relationship is essential for optimizing the design and deployment of AI systems in health care, ensuring that they provide reliable and useful information to users. Moreover, as AI continues to evolve, developing best practices for prompt formulation could enhance the overall user experience and effectiveness of AI‐driven health care services.

This study aims to investigate the impact of prompt construction on the quality of AI chatbot responses, specifically within the context of head and neck surgery.

## Materials and Methods

This study was conducted as part of an international collaborative research project involving young researchers from the International Federation of Otorhinolaryngology Societies and the Italian Society of Maxillofacial Surgery. The consortium was established in February 2023, with the purpose of exploring the potential applications, assessing the reliability, and identifying possible risks associated with AI platforms in the field of head and neck surgery. Sixteen researchers from 11 European centers participated in this study. Ethical committee approval was not required for this study as it did not involve any patients.

### Prompt Development

For the specific purpose of developing the prompt to be used in this study, a multidisciplinary team was constituted. This team included 2 head and neck surgeons, a linguist, and a computer engineer. Their objective was to create a prompt that would effectively guide the AI in providing accurate and contextually appropriate responses. The prompt format was developed through multiple rounds of testing and refinement. Initial versions of the prompt were tested using various clinical scenarios provided by the head and neck surgeons. Feedback was collected and analyzed to identify any areas where the AI responses were suboptimal. Adjustments were made to the prompt format to improve clarity, specificity, and relevance. The final prompt structure was validated by applying it across different clinical cases to ensure its effectiveness in generating high‐quality AI responses.

This process resulted in the creation of a prompt format identified by the acronym SMART, which stands for Seeker, Mission, AI Role, Register, and Targeted Question.

- *Seeker*: It refers to the identity and perspective of the user inquiring (ie head and neck surgeon, general practitioner, medical student, and patient). The prompt must communicate the seeker's role and the clinical context to ensure that the AI understands the expertise level and specific needs of the user.
- *Mission*: It defines the purpose of the inquiry. This involves clearly stating the objective or problem that the AI is expected to address. The mission statement ensures that the AI focuses on the most relevant information.
- *AI Role*: It describes the expected role of the AI in the interaction, which may range from providing information to offering clinical advice. Clearly defining the AI's role helps set expectations for the type and depth of the response.
- *Register*: It involves the tone and style of language to be used in the AI's response. The prompt should guide the AI to use language that is appropriate for the target audience, ensuring clarity and avoiding ambiguity.
- *Targeted Question*: It refers to the specific question or query posed to the AI. This question should be direct and focused, designed to elicit a detailed and accurate response.

### Study Design

A separate group of 3 head and neck surgeons, distinct from those involved in the prompt development, was tasked with creating a set of 24 questions. These questions were divided into 3 groups of 8 questions each, covering different types of inquiries: clinical scenarios, theoretical questions, and patient inquiries. The questions encompassed 3 main areas of head and neck surgery: oncology, sinus and nasal surgery, and trauma. The questions were carefully reviewed to eliminate any potential ambiguities, ensuring clarity and precision.

The questions were formulated in a question format suitable for direct input into the chatbot. Additionally, all questions required the chatbot to provide bibliographic references for the information it utilized in generating responses. The same research group also prepared the contextual information to be included in the SMART prompt (**Table** 1). The “Targeted Question” section of the SMART prompt was then populated with the specific questions devised by the research group.

**Table 1:** SMART Prompt Format

	Clinical scenarios	Theoretical questions	Patient's inquiries
Seeker	I'm a head and neck surgeon with over 15 y of experience, working in a tertiary‐level hospital, and I specialize in (type of surgery relevant to the scenario).	I am a fifth‐year resident specializing in head and neck surgery.	I am a patient.
Mission	I need your advice on how to manage a patient I'm treating.	I need your help to prepare for my final residency exam.	I need medical advice for a problem that has been diagnosed in me.
AI Role	You are the world's leading expert in (type of surgery relevant to the scenario).	You are a full professor of head and neck surgery at the most prestigious university in the world.	The world's leading expert in head and neck surgery.
Register	Correct and specialized scientific language. The information must be based on the most recent and solid scientific evidence. I would like you to also provide me with the bibliographical references from which you draw your information.	Correct and specialized scientific language. The information must be based on the most recent and solid scientific evidence. I would like you to also provide me with the bibliographical references from which you draw your information.	Clear and understandable language even for a nonexpert. The information must be based on the most recent and solid scientific evidence. I would like you to also provide me with the bibliographical references from which you draw your information.
Targeted Question	(Noncontextualized question)	(Noncontextualized question)	(Noncontextualized question)

On July 31, 2024, the uncontextualized question and the question formatted with the SMART prompt were entered into ChatGPT‐4o^17^ by the same researcher. For each question, a new browser window was opened in incognito mode to ensure a fresh session. The responses generated by the AI were collected in a Word document, randomized, and then provided to reviewers for evaluation (Supplemental Table S1, available online).

### Evaluation Protocol

A pool of 3 reviewers, all senior head and neck surgeons with over 15 years of experience in both clinical practice and academic settings, conducted a blind evaluation of the AI‐generated responses to both formats of questions. Each surgeon specializes in complex cases involving head and neck surgery, reconstructive surgery, and advanced sinus disease. Their extensive background includes not only surgical practice but also teaching and publishing in peer‐reviewed journals, ensuring a high level of expertise and familiarity with current best practices in the field. Each reviewer evaluated all responses. Therefore, 3 evaluations were considered for each answer in the analysis. The prompts were not included with the responses, and the reviewers were unaware of the study's nature. During the evaluation process, reviewers were specifically instructed to verify the authenticity of references provided by ChatGPT. The assessment was performed using the Quality Analysis of Medical Artificial Intelligence instrument (QAMAI), a validated tool designed to evaluate the quality of health care information provided by AI chatbots.^18^ The QAMAI instrument assesses various aspects of the information quality, including accuracy, clarity, relevance, completeness, source quality, and usefulness. Each dimension is scored on a scale from 1 to 5, with the total QAMAI score being the sum of these individual scores, allowing a maximum possible score of 30 for each response.

### Statistical Analysis

The statistical analysis was conducted by a blinded researcher using Jamovi software version 2.3.18.0, a freeware and open statistical software available online at www.jamovi.org.^19^ Descriptive statistics for quantitative variables are reported as median (interquartile range [IQR]) or as mean ± standard deviation. The difference between the QAMAI scores obtained from the responses to the 2 question formats was evaluated using the Wilcoxon signed‐rank test for paired samples. In all cases, the level of statistical significance was set at *P* < .05 with a 95% confidence interval.

## Results

Overall, the questions formulated using the SMART prompt yielded significantly higher quality responses compared to those that were not contextualized (QAMAI score 24 [IQR 21.8‐25] vs 27.5 [IQR 25‐29], *P* < .001). The scores were significantly better across all evaluated items: accuracy (4 [IQR 3.75‐4] vs 4.5 [IQR 4‐5], *P* < .001), clarity (4 [IQR 4‐5] vs 5 [IQR 4‐5], *P* = .002), relevance (4 [IQR 3‐4] vs 4.5 [IQR 4‐5], *P* < .001), completeness (4 [IQR 3‐4] vs 5 [IQR 4‐5], *P* < .001), and usefulness (4 [IQR 3.75‐4] vs 5 [IQR 4‐5], *P* < .001).

When analyzing the different types of questions, clinical scenarios presented using the SMART prompt achieved significantly higher QAMAI scores compared to noncontextualized questions (QAMAI score 25 [IQR 23.3‐25.3] vs 28 [IQR 26‐29], *P *< .001). All individual items reported significantly better scores, except for the quality of sources (4 [IQR 4‐5] vs 4 [IQR 4‐5], *P *< .256) (**Table** 2).

**Table 2:** Results of the Assessment of the Quality of the Responses

	Noncontextualized prompt	SMART prompt	
	Median [IQR]	Median [IQR]	*P* value
Clinical scenarios			
Accuracy	4 [4‐5]	5 [4‐5]	<.001
Clarity	4 [4‐5]	5 [5‐5]	.013
Relevance	4 [4‐4]	5 [4‐5]	.001
Completeness	4 [3‐4]	5 [4‐5]	<.001
Sources	4 [4‐5]	4 [4‐5]	.256
Usefulness	4 [3.75‐4]	5 [4‐5]	<.001
QUAMAI score	25 [23.3‐25.3]	28 [26‐29]	<.001
Theoretical questions			
Accuracy	4 [3‐4.25]	4 [4‐5]	.013
Clarity	4 [4‐5]	5 [4‐5]	.187
Relevance	4 [3‐5]	4 [3.75‐5]	.495
Completeness	4 [3‐5]	4 [4‐5]	.181
Sources	2 [2‐3]	4 [4‐4]	<.001
Usefulness	4 [3‐4.25]	4 [4‐5]	.113
QUAMAI score	23 [17.8‐25.3]	25 [23‐28]	.011
Patient's questions			
Accuracy	4 [4‐4]	5 [4‐5]	.003
Clarity	4.5 [4‐5]	5 [4‐5]	.182
Relevance	4 [3.75‐4]	4.5 [4‐5]	.002
Completeness	4 [3.75‐4]	4.5 [4‐5]	.004
Sources	4 [3‐4]	4 [4‐5]	.006
Usefulness	4 [4‐4]	5 [4‐5]	.011
QUAMAI score	24 [23‐25]	28 [25‐29.3]	.001

Regarding theoretical questions, the responses obtained using the SMART prompt were significantly better (QAMAI score 23 [IQR 17.8‐25.3] vs 25 [IQR 23‐28], *P *< .011). In terms of individual items, the questions submitted with the SMART prompt were significantly more accurate (4 [IQR 3‐4.25] vs 4 [IQR 4‐5], *P *= .013) and cited significantly more reliable sources (2 [IQR 2‐3] vs 4 [IQR 4‐4], *P *< .001). The other items did not show significant differences between the 2 formats (**Table** 2).

For patient inquiries, ChatGPT‐4o performed significantly better when using the SMART prompt compared to noncontextualized prompts (QAMAI score 24 [IQR 23‐25] vs 28 [IQR 25‐29.3], *P *= .001). In the individual items, the AI achieved significantly better scores across all categories when using the SMART prompt, except for clarity (4.5 [IQR 4‐5] vs 5 [IQR 4‐5], *P *= .182) (**Table** 2).

Finally, of the 109 bibliographical references produced by the answers to the noncontextualized prompt, 19 (17.4%) were found to be fabricated. The answers to the questions formulated with the SMART prompt instead provided 85 bibliographical references,^10^ 11.8% of which were found to be nonexistent.

## Discussion

The integration of AI in medicine holds the potential for revolutionizing patient care, clinical decision‐making, and medical education.^20^, ^21^, ^22^ AI systems, such as chatbots, can process vast amounts of data quickly, provide tailored information, and enhance accessibility to medical knowledge. However, the use of AI also carries significant risks, including the potential for disseminating inaccurate information, over‐reliance on automated systems, and challenges in ensuring patient privacy and data security.^23^, ^24^ Balancing these potentials with the associated risks is crucial as AI becomes increasingly embedded in health care practices.^25^

The findings of this study highlight the critical role that prompt construction plays in enhancing the quality of AI chatbot responses, particularly within the context of head and neck surgery. The introduction of the SMART prompt format has demonstrated a significant improvement in the overall performance of AI‐driven chatbots, as evidenced by the superior QAMAI scores across its various dimensions. One of the most striking outcomes of this study is the clear benefit of providing detailed and structured contextual information through the SMART prompt format allowing the AI to generate responses that are more precise and tailored to the specific needs of the user. The ability of the AI to deliver more accurate and relevant responses in these complex scenarios underscores the importance of a well‐structured prompt that encapsulates the necessary context for effective interaction.

In clinical scenarios, the responses generated with the SMART prompt not only delve deeper into the details of the case but also provide a hierarchical order of possible treatments, including specific drug dosages and a more critical analysis of the scenarios. The AI considers all the provided information to offer more precise and appropriate therapeutic recommendations. This detailed approach is crucial in ensuring that the AI can assist health care professionals in making well‐informed clinical decisions. It is important to acknowledge that the AI responses generated without the SMART prompt performed well across most evaluation scales. This baseline quality is consistent with previous studies highlighting large language models' ability to produce generally accurate and relevant content. However, our analysis revealed that the SMART prompt added an extra layer of specificity and contextual relevance that nonprompted responses sometimes lacked. For example, in cases of odontogenic sinusitis, responses without the SMART prompt recommended broad‐spectrum antibiotics in general terms, while the SMART‐prompted responses specified antibiotics and tailored durations depending on clinical response (Supplemental Table S1, available online—Clinical scenarios question 1). Another clinical scenario highlighted these improvements: when analyzing a case of HPV‐positive T2N0M0 oropharyngeal squamous cell carcinoma, the noncontextualized response merely listed possible treatment options (surgery, radiotherapy, and chemoradiotherapy) without identifying a recommended first‐line treatment. In contrast, the SMART‐prompted response provided a detailed examination of factors guiding the choice between radiotherapy and surgery, specifically recommending radiotherapy as the preferred initial option for such cases (Supplemental Table S1, available online—Clinical scenarios question 2).

This study also reveals that the benefits of the SMART prompt are not uniform across all types of questions. While clinical scenarios and patient inquiries showed marked improvements, the difference in performance for theoretical questions, although statistically significant, was less pronounced. This could suggest that the inherent nature of theoretical questions, which may require less contextualization, limits the degree to which prompt formatting can influence AI performance. However, even in these cases, the SMART prompt led to better source quality, indicating that the structured approach encourages the AI to reference more reliable and relevant information, a crucial factor in scientific and educational contexts.^26^, ^27^

In patient inquiries, the SMART prompt also proved effective, enabling the AI to adjust the context appropriately by providing the correct information in an accessible language. This is particularly important as more patients are likely to turn to AI for health care information in the future. Ensuring that patients can obtain the most reliable information will be essential. As AI continues to play an increasingly prominent role in patient care and medical education, the importance of optimizing AI–human interactions cannot be overstated. The SMART prompt format provides a practical framework for enhancing the efficacy of AI tools, ensuring that they can deliver high‐quality, contextually appropriate information.

In the literature, high rates of fabricated references have been reported with both ChatGPT‐3.5 and ChatGPT‐4 models.^26^, ^27^ This phenomenon is likely due to the way generative AI models, such as ChatGPT, construct language. Unlike traditional retrieval‐based systems, generative models produce responses by predicting the next word in a sequence based on training data, rather than accessing an external database of verified sources. When tasked with generating a bibliographic reference—a complex and structured type of text—these models are prone to mixing elements from different references they have encountered during training, resulting in plausible yet entirely fictitious citations. In our study, we observed fabricated references in 17.4% of responses generated with noncontextualized prompts and 11.8% with the SMART prompt, which are both lower than the rates commonly reported in the literature for both ChatGPT‐3.5 and earlier versions of ChatGPT‐4.^26^, ^27^ We did not conduct a comparative analysis to determine the significance of this difference, as the types of references generated by the 2 prompt structures were notably different. Noncontextualized prompts led the AI to provide a higher number of references to institutional websites and clinical guidelines, while the SMART prompt encouraged more references to specific scientific articles. It is probable that the AI model is more likely to produce hallucinated references when citing scientific articles due to the greater linguistic complexity involved in crafting accurate citations for such sources, as opposed to referencing websites or guidelines. Despite the improvements achieved with the SMART prompt, a 10% rate of fabricated references remains unacceptably high for broad clinical applicability, necessitating meticulous verification of every cited reference.

An additional consideration is the potential for large language models to improve response quality over iterative interactions with a user. While such models can refine their outputs within a single session by adapting to follow‐up questions, this adaptability is constrained by the lack of persistent memory across different sessions, which prevents cumulative learning over time. In our study, we focused on single interactions to standardize the comparison between noncontextualized and SMART‐prompted responses. Although repeated, nonprompted interactions might yield some improvement in response quality, the SMART prompt structure provides an immediate and consistent framework for generating specialized, high‐quality answers without requiring iterative adjustments. This underscores the utility of structured prompting in settings where time and response accuracy are critical, such as in medical practice.

While this study provides robust evidence supporting the utility of the SMART prompt, there are some limitations that should be acknowledged. The study was conducted using a specific AI platform (ChatGPT‐4o); while the results are promising, they may not be fully generalizable to other AI systems. Additionally, the study focused on a relatively narrow field of medicine; future research should explore the applicability of the SMART prompt format across different medical specialties and AI platforms. Another limitation to consider is that AI models, such as the one used in this study, are continually being updated and improved. The findings presented here reflect the capabilities of the model at a specific point in time. Future updates to the model may alter its performance and response quality, potentially affecting the relevance of our results in the long term. Thus, these findings should be interpreted as representative of the model's capabilities at the time of the study, rather than a permanent assessment of its performance.

Further research is also needed to refine the SMART prompt format and explore its potential integration into AI systems as a standard feature. This could involve developing automated tools that assist users in constructing effective prompts, thereby democratizing access to high‐quality AI interactions across a broader range of health care professionals.

## Conclusions

In conclusion, this study demonstrates that the quality of AI‐generated responses in health care can be significantly enhanced through the use of a structured, context‐rich prompt format such as SMART. This approach not only improves the accuracy and relevance of the information provided but also contributes to more reliable and effective use of AI in clinical practice. As AI continues to evolve, the development of best practices for prompt formulation will be essential to maximize the potential of these technologies in improving patient care and supporting medical professionals.

## Author Contributions

**Luigi Angelo Vaira**, conceptualization of the work, development of the methodology, data curation, writing the original draft, writing the final draft, and final approval; **Jerome R. Lechien**, conceptualization of the work, development of the methodology, data curation, writing the original draft, writing the final draft, and final approval; **Vincenzo Abbate**, data collection, data curation, revision of the original and final draft, and final approval; **Guido Gabriele**, data collection, data curation, revision of the original and final draft, and final approval; **Andrea Frosolini**, development of the methodology, data curation, revision of the original and final draft, and final approval; **Andrea De Vito**, development of the methodology, data curation, revision of the original and final draft, and final approval; **Antonino Maniaci**, literature review, data curation, revision of the original and final draft, and final approval; **Miguel Mayo‐Yáñez**, literature review, data curation, revision of the original and final draft, and final approval; **Paolo Boscolo‐Rizzo**, literature review, data curation, revision of the original and final draft, and final approval; **Alberto Maria Saibene**, development of the methodology, data curation, revision of the original and final draft, and final approval; **Fabio Maglitto**, development of the methodology, data curation, revision of the original and final draft, and final approval; **Giovanni Salzano**, data collection, data curation, revision of the original and final draft, and final approval; **Gianluigi Califano**, statistical analysis, data curation, revision of the original and final draft, and final approval; **Stefania Troise**, development of the methodology, data curation, revision of the original and final draft, and final approval; **Carlos Miguel Chiesa‐Estomba**, development of the methodology, data curation, revision of the original and final draft, and final approval; **Giacomo De Riu**, supervision, conceptualization of the work, development of the methodology, data curation, writing the original draft, writing the final draft, and final approval.

## Disclosure

### Competing interests

None.

### Funding source

This work has been developed within the framework of the project e.INS‐Ecosystem of Innovation for Next Generation Sardinia (cod. ECS 00000038) funded by the Italian Ministry for Research and Education (MUR) under the National Recovery and Resilience Plan (NRRP) MISSION 4 COMPONENT 2, “From research to business” (INVESTMENT 1.5), “Creation and strengthening of Ecosystems of innovation,” and construction of “Territorial R&D Leaders” (CUP J83C21000320007).

## Supporting information

Supporting information.
