# Supplementary material for: Enhancing AI Chatbot Responses in Health Care: The SMART Prompt Structure in Head and Neck Surgery
Source: OTO Open. 2025 Jan 16;9(1):e70075. doi: 10.1002/oto2.70075 (PMC11736147; doi:10.1002/oto2.70075)
Supplement: Supplementary file 1 — Supporting information. [file OTO2-9-e70075-s001.docx]

| **Supplementary table 1. Questions and answers given by ChatGPT-4.** | | | | | |
| --- | --- | --- | --- | --- | --- |
| **Category** | **Question number** | **Non-contextualized question** | **Answer** | **SMART prompt** | **Answer** |
| Clinical scenario | 1 | A 42 years old male with no other significant medical history has been experiencing right facial pain, foul nasal discharge and nasal obstruction for 3 months. The computed tomography shows complete opacification of the right maxillary and ethmoid sinus with cortical bone focal erosion over the second upper right molar. The endoscopy shows purulent discharge in the right nasal cavity. What is the possible diagnosis? How the patient could be managed from a clinical and surgical point of view? Provide detailed diagnosis and - if needed - drugs and surgical procedures the head and neck surgeon would employ in this case? Please provide me with the bibliographical references from which you draw your information. | Given the clinical presentation of chronic right facial pain, foul nasal discharge, nasal obstruction, and the findings on computed tomography (CT) and endoscopy, the most likely diagnosis is **chronic rhinosinusitis with a possible odontogenic origin**. The presence of cortical bone erosion over the second upper right molar suggests that the infection could have originated from or been exacerbated by a dental issue.  **Possible Diagnosis**   - **Chronic Rhinosinusitis (CRS)** with potential odontogenic sinusitis.   **Diagnostic Approach**   1. **Clinical History and Physical Examination**:    - Detailed history focusing on the duration and characteristics of symptoms.    - Examination of the nasal cavity and oropharynx. 2. **Imaging**:    - **Computed Tomography (CT)** of the sinuses is the gold standard for diagnosing sinusitis and assessing the extent of sinus involvement.    - Evaluation for dental pathology that might contribute to the sinus disease. 3. **Endoscopy**:    - Nasal endoscopy to visualize the purulent discharge and identify anatomical abnormalities or polyps. 4. **Dental Evaluation**:    - Examination by a dentist or oral surgeon to evaluate for any dental infections or periapical abscesses that could be contributing to the sinusitis.   **Management**  **Medical Management**   1. **Antibiotics**:    - Broad-spectrum antibiotics tailored to the likely pathogens (e.g., Amoxicillin-Clavulanate, Clindamycin, or Metronidazole) based on culture and sensitivity if available.    - Duration typically 3-6 weeks depending on response. 2. **Nasal Corticosteroids**:    - Topical nasal corticosteroids (e.g., Fluticasone, Mometasone) to reduce inflammation. 3. **Saline Irrigations**:    - Regular saline nasal irrigations to clear purulent discharge and improve mucociliary clearance. 4. **Pain Management**:    - Analgesics such as acetaminophen or nonsteroidal anti-inflammatory drugs (NSAIDs) for pain relief.   **Surgical Management**   1. **Functional Endoscopic Sinus Surgery (FESS)**:    - Indicated if there is a lack of improvement with medical management or if there is significant anatomical obstruction.    - The surgery involves the removal of diseased tissue, drainage of sinuses, and correction of any anatomical blockages.    - Specifically, the maxillary and ethmoid sinuses would be targeted for drainage and debridement. 2. **Addressing Dental Origin**:    - Extraction or root canal treatment of the affected tooth if odontogenic sinusitis is confirmed.    - Collaboration with an oral surgeon or dentist for simultaneous management of dental pathology.   **References**   1. **Rosenfeld, R. M., Piccirillo, J. F., Chandrasekhar, S. S., et al. (2015). Clinical practice guideline (update): adult sinusitis. Otolaryngology–Head and Neck Surgery, 152(2_suppl), S1-S39.**    - This guideline provides a comprehensive approach to the diagnosis and management of sinusitis, including medical and surgical treatment options. 2. **Fokkens, W. J., Lund, V. J., Mullol, J., et al. (2020). European position paper on rhinosinusitis and nasal polyps 2020. Rhinology, 58(Suppl S29), 1-464.**    - This position paper details the diagnostic criteria, treatment strategies, and management of chronic rhinosinusitis and related conditions. 3. **Brook, I. (2004). Microbiology and antibicrobial management of sinusitis. Otolaryngol Clin North Am, 37(2), 253-266.**    - This article discusses the microbiology of sinusitis and the appropriate antibiotic management strategies. 4. **Mathews, M. S. (2011). Management of odontogenic sinusitis. Otolaryngologic Clinics of North America, 44(3), 539-549.**    - This review focuses on the diagnosis and management of sinusitis of dental origin, including the role of dental procedures in treatment.   The combination of medical and surgical treatment tailored to the patient's specific condition, with a focus on addressing any underlying dental issues, will provide the best outcome in this case. | Seeker: I'm a head and neck surgeon with over 15 years of experience, working in a tertiary-level hospital, and I specialize in nasal and paranasal sinus surgery.  Mission: I need your advice on how to manage a patient I'm treating  AI Role: You are the world's leading expert in nasal and paranasal sinus surgery.  Register: correct and specialized scientific language. The information must be based on the most recent and solid scientific evidence. I would like you to also provide me with the bibliographical references from which you draw your information  Targeted question: A 42 years old male with no other significant medical history has been experiencing right facial pain, foul nasal discharge and nasal obstruction for 3 months. The computed tomography shows complete opacification of the right maxillary and ethmoid sinus with cortical bone focal erosion over the second upper right molar. The endoscopy shows purulent discharge in the right nasal cavity. What is the possible diagnosis? How the patient could be managed from a clinical and surgical point of view? Provide detailed diagnosis and - if needed - drugs and surgical procedures the head and neck surgeon would employ in this case and provide bibliographical references to your information. | **Possible Diagnosis**  Based on the provided clinical presentation and imaging findings, the most likely diagnosis for this patient is **chronic rhinosinusitis (CRS) with nasal polyps (CRSwNP) complicated by odontogenic sinusitis**. The presence of cortical bone erosion over the second upper right molar and the purulent discharge strongly suggests an odontogenic origin of the sinusitis.  **Diagnosis**   1. **Chronic Rhinosinusitis (CRS)**:    - **Diagnostic Criteria**: Symptoms persisting for more than 12 weeks, including nasal obstruction, facial pain/pressure, and purulent nasal discharge.    - **Imaging**: CT scan showing opacification of the maxillary and ethmoid sinuses.    - **Endoscopy**: Visualization of purulent discharge in the nasal cavity. 2. **Odontogenic Sinusitis**:    - **Etiology**: Dental infections, particularly those involving the upper molars, can extend into the maxillary sinuses.    - **Imaging**: CT findings of sinus opacification with focal bone erosion near dental roots.   **Management**  **Medical Management**   1. **Antibiotic Therapy**:    - Given the purulent nature of the discharge and evidence of bacterial infection, broad-spectrum antibiotics are warranted.    - **First-line antibiotic**: Amoxicillin-clavulanate (875 mg/125 mg twice daily for 10-14 days).    - **Alternative in penicillin-allergic patients**: Doxycycline (100 mg twice daily) or clindamycin (300 mg four times daily). 2. **Nasal Corticosteroids**:    - To reduce inflammation and polyp size.    - **Example**: Mometasone furoate nasal spray (two sprays in each nostril once daily). 3. **Saline Irrigation**:    - To help clear nasal passages and reduce symptoms.    - **Example**: Isotonic saline nasal irrigation twice daily.   **Surgical Management**   1. **Functional Endoscopic Sinus Surgery (FESS)**:    - Indicated if there is no response to medical therapy or in the presence of complications such as bone erosion.    - Goals: To remove obstructive tissue, drain the sinuses, and restore normal sinus ventilation.    - Procedure: Involves the endoscopic removal of polyps, debris, and infected tissue from the sinuses. The ostia of the sinuses are widened to facilitate drainage. 2. **Dental Intervention**:    - Addressing the odontogenic source of infection is crucial.    - This may include dental extraction or root canal treatment of the affected tooth. 3. **Post-Operative Care**:    - Continuation of nasal corticosteroids and saline irrigation.    - Follow-up endoscopic examination to monitor healing and detect any recurrence.   **Bibliographical References**   1. **Fokkens WJ, Lund VJ, Hopkins C, et al.** "European Position Paper on Rhinosinusitis and Nasal Polyps 2020." Rhinology. 2020 Feb;58(Suppl S29):1-464. 2. **Rosenfeld RM, Piccirillo JF, Chandrasekhar SS, et al.** "Clinical Practice Guideline (Update): Adult Sinusitis." Otolaryngology–Head and Neck Surgery. 2015 Apr;152(2_suppl)   .   1. **Brook I.** "Microbiology and antimicrobial management of sinusitis." Otolarungology Clinics North American. 2004 Apr;37(2):253-266. 2. **Kennedy DW, Adappa ND.** "Endoscopic Sinus Surgery: Evolution and Technical Innovations." Journal of Laryngology and Otology. 2018;128(4):225-232.   These references encompass the most recent and comprehensive guidelines and research on the diagnosis and management of chronic rhinosinusitis and odontogenic sinusitis, supporting the clinical and surgical recommendations provided. |
|  | QAMAI Scores: Reviewer 1: 25; Reviewer 2: 26; Reviewer 3: 29 | | | QAMAI Scores: Reviewer 1: 29; Reviewer 2: 30; Reviewer 3: 28 | |
| Clinical scenario | 2 | A patient with a lesion of the lateral wall of the oropharynx underwent a biopsy with the result of moderately differentiated squamous cell carcinoma. HPV DNA research was positive. The contrast MRI staged the lesion as T2N0M0. What is the treatment of choice in this case? Please provide me with the bibliographical references from which you draw your information. | For a patient with moderately differentiated squamous cell carcinoma of the oropharynx, HPV-positive, and staged as T2N0M0, the treatment of choice typically involves:   1. **Primary Surgery or Radiation Therapy**: Both surgery and radiation therapy are effective for T2N0M0 oropharyngeal cancer. The choice between the two depends on various factors, including the exact location and extent of the tumor, patient comorbidities, and preferences.    - **Surgery**: Transoral robotic surgery (TORS) or transoral laser microsurgery (TLM) are minimally invasive surgical options that can be considered.    - **Radiation Therapy**: If surgery is not feasible or preferred, definitive radiation therapy can be an effective alternative. 2. **Consideration of Chemotherapy**: For HPV-positive oropharyngeal cancer, concurrent chemoradiotherapy is often reserved for more advanced stages. In T2N0M0, chemotherapy is not typically indicated unless there are high-risk features. 3. **Follow-up and Monitoring**: Regular follow-up with imaging and physical examinations to monitor for recurrence or metastasis is crucial.   The specific treatment plan should be personalized based on multidisciplinary team discussions and the patient's overall health and preferences.  Here are some bibliographical references that support the information provided:   1. **National Comprehensive Cancer Network (NCCN) Guidelines**:    - National Comprehensive Cancer Network. "NCCN Clinical Practice Guidelines in Oncology: Head and Neck Cancers." Available at: NCCN. (Accessed August 2024). 2. **American Cancer Society**:    - American Cancer Society. "Treatment of Oropharyngeal Cancer by Stage." Available at: American Cancer Society. (Accessed August 2024). 3. **Journal of Clinical Oncology**:    - Ang, K.K., Harris, J., Wheeler, R., et al. "Human papillomavirus and survival of patients with oropharyngeal cancer." *New England Journal of Medicine*, 363(1), 24-35, 2010. DOI: 10.1056/NEJMoa0912217. 4. **Clinical Practice Guidelines for the Diagnosis and Management of Squamous Cell Carcinoma of the Head and Neck**:    - Pfister, D.G., Spencer, S., Adelstein, D., et al. "Head and Neck Cancers, Version 1.2022, NCCN Clinical Practice Guidelines in Oncology." *Journal of the National Comprehensive Cancer Network (JNCCN)*, 20(4), 2022. DOI: 10.6004/jnccn.2022.0015.   These sources provide comprehensive guidelines and evidence-based recommendations for the management of oropharyngeal squamous cell carcinoma, including the role of HPV status and staging in treatment decisions. | Seeker: I'm a head and neck surgeon with over 15 years of experience, working in a tertiary-level hospital, and I specialize in oncologic surgery.  Mission: I need your advice on how to manage a patient I'm treating  AI Role: You are the world's leading expert in oncologic surgery.  Register: correct and specialized scientific language. The information must be based on the most recent and solid scientific evidence. I would like you to also provide me with the bibliographical references from which you draw your information  Targeted question: A patient with a lesion of the lateral wall of the oropharynx underwent a biopsy with the result of moderately differentiated squamous cell carcinoma. HPV DNA research was positive. The contrast MRI staged the lesion as T2N0M0. What is the treatment of choice in this case? | In the management of a patient with T2N0M0 moderately differentiated squamous cell carcinoma (SCC) of the lateral wall of the oropharynx with a positive HPV status, the treatment strategy should be determined based on current evidence and guidelines. Here is an outline of the treatment options and considerations:  **Treatment Strategy for HPV-Positive T2N0M0 Oropharyngeal SCC**   1. **Primary Treatment Modalities:**    - **Radiotherapy (RT):** For early-stage oropharyngeal cancers (T1-T2), definitive radiation therapy alone is a standard treatment option. Intensity-modulated radiation therapy (IMRT) is preferred due to its precision and reduced toxicity to surrounding tissues.    - **Surgery:** Transoral robotic surgery (TORS) or transoral laser microsurgery (TLM) can be considered for resectable T2 tumors. Surgical resection is often followed by postoperative radiation therapy (PORT) based on the pathological findings, such as positive margins or extracapsular spread. 2. **Radiotherapy vs. Surgery:**    - The choice between primary surgery and radiotherapy depends on various factors, including tumor accessibility, patient's functional status, potential morbidity of treatment, and patient's preference.    - Given the positive HPV status, these tumors tend to respond better to radiation and have a better prognosis, making definitive RT a highly viable option. 3. **Adjuvant Therapy:**    - **Postoperative Radiation Therapy (PORT):** If surgery is chosen, adjuvant RT is indicated in cases with adverse features such as perineural invasion, lymphovascular invasion, or close/positive margins.    - **Chemoradiotherapy (CRT):** Concurrent chemoradiotherapy might be considered for patients with high-risk features, although for T2N0M0 with a favorable HPV profile, RT alone is often sufficient. 4. **HPV-Positive SCC Considerations:**    - HPV-positive oropharyngeal cancers have distinct clinical behavior, with better responses to treatment and improved survival rates compared to HPV-negative tumors.    - De-escalation strategies (e.g., reduced radiation dose) are currently being investigated in clinical trials for HPV-positive patients to minimize long-term treatment-related toxicities while maintaining high cure rates.   **Recommended Treatment Approach:**  Based on the current evidence and guidelines, the recommended treatment approach for a T2N0M0 moderately differentiated HPV-positive squamous cell carcinoma of the lateral wall of the oropharynx would be:   - **Definitive Radiation Therapy (IMRT):** This is typically the treatment of choice, given the tumor's stage and HPV-positive status, which predicts a good response to RT. - **Surgical Approach (TORS or TLM):** If the lesion is accessible and the patient is a suitable candidate for surgery, TORS or TLM followed by adjuvant RT (if indicated) could be considered.   **Key References:**   1. **National Comprehensive Cancer Network (NCCN) Guidelines: Head and Neck Cancers, Version 1.2024.** 2. **Ang, K.K., Harris, J., Wheeler, R., et al. (2010). Human papillomavirus and survival of patients with oropharyngeal cancer. New England Journal of Medicine, 363(1), 24-35.** 3. **Fakhry C, Westra WH, Li S, et al. (2008) Improved survival of patients with human papillomavirus-positive head and neck squamous cell carcinoma in a prospective clinical trial. J Natl Cancer Inst, 100(4), 261-269.** 4. **Mehanna, H., et al. (2019). De-ESCALaTE HPV: A Phase 3 Trial of Radiotherapy Plus Cisplatin Versus Radiotherapy Plus Cetuximab in Low-Risk HPV-Positive Oropharyngeal Cancer. Lancet, 393, 51-60.**   This approach ensures a balance between efficacy and minimization of treatment-related morbidity, tailored to the HPV-positive status of the carcinoma. |
|  |  | QAMAI Scores: Reviewer 1: 25; Reviewer 2: 20; Reviewer 3: 25 | | QAMAI Scores: Reviewer 1: 29; Reviewer 2: 28; Reviewer 3: 30 | |
| Clinical scenario | 3 | An MMA fighter reports noticing the sudden appearance of swelling at the left periorbital area after blowing his nose after a match 7 days ago. The swelling gradually disappeared but for about two days he reports diplopia in upward gaze. What could be the diagnosis? How would the doctor manage the patient from a clinical and surgical point of view? Provide detailed diagnosis and - if needed - drugs and surgical procedures the head and neck surgeon would employ in this case. Please provide me with the bibliographical references from which you draw your information. | The symptoms described by the MMA fighter suggest a possible orbital blowout fracture, specifically involving the orbital floor. This type of injury often occurs due to blunt trauma to the orbit, causing a fracture of the thin bones around the eye, such as the orbital floor or medial wall. The diplopia (double vision) in upward gaze indicates the possible entrapment of the inferior rectus muscle or other orbital contents in the fracture site.  **Diagnosis**   1. **Clinical Examination**:    - **Inspection**: The doctor will inspect for periorbital swelling, ecchymosis, and enophthalmos (sunken eye).    - **Palpation**: To check for crepitus or tenderness around the orbit.    - **Ophthalmic Examination**: Visual acuity, pupillary response, and ocular motility will be assessed.    - **Diplopia Assessment**: Specifically checking for double vision on upward gaze. 2. **Imaging**:    - **CT Scan of the Orbit**: This is the gold standard for diagnosing orbital fractures. It provides detailed images of the bony structures and can show the extent of the fracture, the involvement of the orbital contents, and the presence of any muscle entrapment.   **Management**  **Clinical Management:**   1. **Initial Management**:    - **Cold Compresses**: To reduce swelling.    - **Pain Management**: NSAIDs or acetaminophen for pain relief.    - **Antibiotics**: Prophylactic antibiotics (e.g., amoxicillin-clavulanate) to prevent infection, especially if there is a risk of sinus involvement. 2. **Observation**:    - Small, non-displaced fractures without significant symptoms might be managed conservatively with observation and follow-up.   **Surgical Management:**   1. **Indications for Surgery**:    - Persistent diplopia in primary or significant gaze positions.    - Significant enophthalmos (>2 mm).    - Large fracture (>50% of the orbital floor).    - Evidence of muscle or tissue entrapment causing persistent symptoms. 2. **Surgical Procedure**:    - **Orbital Floor Repair**: The goal is to free any entrapped tissues and repair the defect to prevent recurrence of symptoms.    - **Approach**: A transconjunctival or transcutaneous approach can be used to access the orbital floor.    - **Materials**: The fracture is repaired using materials such as titanium mesh, porous polyethylene (Medpor), or autologous bone grafts. 3. **Postoperative Care**:    - **Antibiotics**: Continue antibiotics postoperatively to prevent infection.    - **Steroids**: Short course of corticosteroids to reduce inflammation and edema.    - **Follow-Up**: Regular follow-up to monitor healing and ensure the resolution of symptoms.   **References**   - **American Academy of Ophthalmology**. (2020). *Orbital Fracture Repair*. Retrieved from [AAO.org](https://www.aao.org). - **Hink EM, Wei LA, Durairaj VD**. (2014). "Clinical features and treatment of isolated orbital floor fractures". *Ophthalmic Plast Reconstr Surg*. 30(5):421-426. - **Dua HS, King AJ, Joseph A**. (2004). *A Manual of Systematic Eyelid and Orbital Surgery*. Oxford University Press.   These references provide a comprehensive overview of the clinical features, diagnosis, and management options for orbital blowout fractures, ensuring the information is evidence-based and aligned with current medical standards. | Seeker: I'm a head and neck surgeon with over 15 years of experience, working in a tertiary-level hospital, and I specialize in trauma surgery  Mission: I need your advice on how to manage a patient I'm treating.  AI Role: You are the world's leading expert in trauma surgery  Register: correct and specialized scientific language. The information must be based on the most recent and solid scientific evidence. I would like you to also provide me with the bibliographical references from which you draw your information  Targeted question: An MMA fighter reports noticing the sudden appearance of swelling at the left periorbital area after blowing his nose after a match 7 days ago. The swelling gradually disappeared but for about two days he reports diplopia in upward gaze. What could be the diagnosis? How would the doctor manage the patient from a clinical and surgical point of view? Provide detailed diagnosis and - if needed - drugs and surgical procedures the head and neck surgeon would employ in this case. | The symptoms described, specifically the sudden onset of periorbital swelling following nose-blowing and subsequent diplopia in upward gaze, strongly suggest a **blowout fracture of the orbit**, most likely involving the orbital floor or medial wall. This type of injury can occur in situations like an MMA fight where there is significant blunt trauma to the orbital region.  **Pathophysiology:**  A blowout fracture occurs when a direct impact to the orbit results in a sudden increase in intraorbital pressure. This causes the orbital contents to herniate through the weakest point of the orbital wall, commonly the floor (maxillary sinus) or the medial wall (ethmoid sinus). The valsalva maneuver (e.g., nose blowing) can exacerbate this by forcing air into the orbital cavity through the fracture site, leading to periorbital emphysema and swelling.  **Clinical Examination and Diagnosis:**   1. **History and Physical Examination**:    - Detailed history of the trauma, emphasizing the mechanism of injury.    - Inspection for periorbital ecchymosis, swelling, and step-offs in the orbital rim.    - Palpation for crepitus, suggesting subcutaneous emphysema.    - Ophthalmologic examination to assess visual acuity, diplopia, globe position, and extraocular movements. Diplopia on upward gaze typically indicates entrapment of the inferior rectus muscle or inferior oblique muscle.    - Assessment for enophthalmos (posterior displacement of the globe), which can occur due to herniation of orbital contents. 2. **Imaging**:    - **CT Scan**: High-resolution CT of the orbits is the gold standard. Axial and coronal views will reveal the extent of the fracture, displacement of bony fragments, and herniation of orbital contents. Soft tissue windows will help assess muscle entrapment.   **Management:**  **Non-Surgical Management:**   - **Observation**: If there is minimal diplopia, no significant enophthalmos, and no muscle entrapment, conservative management with ice packs, decongestants, and avoiding nose blowing may suffice. Oral corticosteroids may be used to reduce swelling.   **Surgical Management:**  Surgical intervention is indicated in cases of significant enophthalmos (>2 mm), persistent diplopia due to muscle entrapment, large orbital floor defect, or cosmetic deformity.   1. **Timing of Surgery**:    - Surgery is ideally performed within 1-2 weeks to prevent fibrosis and permanent muscle damage but can be delayed if severe orbital edema is present. 2. **Surgical Approach**:    - **Transconjunctival Approach**: Provides good access to the orbital floor with minimal visible scarring.    - **Subciliary or Infraorbital Approaches**: These may be used but have higher risk of visible scars and ectropion. 3. **Procedure**:    - **Reduction and Fixation**: Reduction of herniated orbital contents and repair of the fracture using implants (e.g., titanium mesh, absorbable plates) to reconstruct the orbital floor and provide support.    - **Endoscopic Approach**: In some cases, especially with medial wall fractures, an endoscopic transnasal approach may be utilized. 4. **Postoperative Care**:    - Prophylactic antibiotics to prevent sinusitis.    - Nasal decongestants and advice against nose blowing.    - Follow-up to monitor for resolution of diplopia and assess for any postoperative complications.   **Medications:**   - **Antibiotics**: Prophylactic antibiotics, such as amoxicillin-clavulanate, to prevent infection. - **Steroids**: Oral prednisone to reduce inflammation and edema. - **Analgesics**: NSAIDs or acetaminophen for pain management. - **Decongestants**: Pseudoephedrine to reduce nasal congestion and avoid exacerbating orbital emphysema.   **References:**   1. **Burnstine MA. Clinical recommendations for repair of orbital facial fractures. *Curr Opin Ophthalmol*. 2003;14(5):236-240.** 2. **Smith B, Regan WF Jr. Blow-out fracture of the orbit; mechanism and correction of internal orbital fracture. *Am J Ophthalmol*. 1957;44(6):733-739.** 3. **Kontio R, Lindqvist C. Management of orbital fractures. *Oral Maxillofac Surg Clin North Am*. 2009;21(2):209-vi.**   This information should provide a comprehensive framework for the diagnosis and management of this patient's condition. Further individualized assessment and management should be based on the patient's specific clinical findings and imaging results. |
|  |  | QAMAI Scores: Reviewer 1: 24; Reviewer 2: 27; Reviewer 3: 29 | | QAMAI Scores: Reviewer 1: 24; Reviewer 2: 28; Reviewer 3: 28 | |
| Clinical scenario | 4 | A patient underwent a left lateral neck dissection for tongue cancer. Four days after the operation, the drain collected 500 mL of clear, serous fluid in 24 hours. What could be the diagnosis? What are the possible treatments? Please provide me with the bibliographical references from which you draw your information. | The accumulation of 500 mL of clear, serous fluid in the drain four days post-operation suggests the presence of a **chyle leak** or **chylous fistula**. This condition occurs when the thoracic duct or one of its branches is injured during neck surgery, leading to the leakage of lymphatic fluid (chyle).  **Possible Treatments:**  The treatment of a chyle leak varies depending on the severity and persistence of the leak. Here are some potential approaches:   1. **Conservative Management**:    - **Dietary Modifications**: Implementing a low-fat diet or a medium-chain triglyceride (MCT) diet can reduce the flow of chyle.    - **Total Parenteral Nutrition (TPN)**: Temporarily eliminating oral intake and providing nutrition intravenously to reduce lymph production.    - **Octreotide**: A somatostatin analog that can reduce lymphatic flow and has been used successfully to manage chyle leaks. 2. **Interventional Procedures**:    - **Percutaneous Thoracic Duct Embolization**: A minimally invasive procedure to occlude the thoracic duct and stop the leak.    - **Lymphangiography**: This imaging technique can be both diagnostic and therapeutic, sometimes resulting in spontaneous closure of the leak. 3. **Surgical Intervention**:    - **Thoracic Duct Ligation**: If conservative measures fail, surgical ligation of the thoracic duct may be necessary.    - **Direct Repair**: In some cases, direct repair of the leak site during surgery might be required.   **Bibliographic References:**   1. **Bueth M. Surgical management of chyle leakage: Thoracic duct injury and management. Seminars in Thoracic and Cardiovascular Surgery.** 2021;33(3):1023-1029. DOI: 10.1053/j.semtcvs.2021.01.015. 2. **Ilczyszyn A, Ridha H, Durrani AJ. Management of chyle leak post head and neck surgery: Review of current literature.** American Journal of Otolaryngology. 2017;38(6):563-568. DOI: 10.1016/j.amjoto.2017.07.002. 3. **Kranzfelder M, Gertler R, Hapfelmeier A, Friess H, Büchler P. Chylous ascites in surgical oncology: Overview and treatment algorithm.** Cancer Treatment Reviews. 2011;37(5):387-395. DOI: 10.1016/j.ctrv.2011.01.003.   These sources provide comprehensive information on the management and treatment options for chyle leaks post-surgery. | Seeker: I'm a head and neck surgeon with over 15 years of experience, working in a tertiary-level hospital, and I specialize in oncologic surgery  Mission: I need your advice on how to manage a patient I'm treating.  AI Role: You are the world's leading expert in oncologic surgery  Register: correct and specialized scientific language. The information must be based on the most recent and solid scientific evidence. I would like you to also provide me with the bibliographical references from which you draw your information  Targeted question: A patient underwent a left lateral neck dissection for tongue cancer. Four days after the operation, the drain collected 500 mL of clear, serous fluid in 24 hours. What could be the diagnosis? What are the possible treatments? | The presentation of a patient with a significant amount of clear, serous fluid accumulation in the drain post-lateral neck dissection for tongue cancer suggests a possible **chyle leak (chylous fistula)**. This condition occurs when the thoracic duct or one of its tributaries is injured during the surgical procedure.  **Diagnosis Confirmation:**   - **Clinical presentation:** Rapid accumulation of a significant amount of milky, odorless fluid. - **Laboratory analysis of the fluid:** Elevated triglyceride levels (>110 mg/dL) and the presence of chylomicrons. - **Imaging:** Lymphangiography or lymphoscintigraphy to localize the site of the leak.   **Management and Treatment:**  The management of chyle leak can be divided into conservative and surgical approaches, depending on the severity and response to initial treatments.  **Conservative Management:**   1. **Dietary Modification:**    - Initiate a **medium-chain triglyceride (MCT) diet** or a **fat-free diet** to reduce the production of chyle.    - In refractory cases, consider **total parenteral nutrition (TPN)** to completely rest the lymphatic system. 2. **Pharmacologic Therapy:**    - Use of **octreotide**, a somatostatin analog, which can reduce lymphatic flow and promote closure of the leak. 3. **Pressure Dressings:**    - Application of a **pressure dressing** to the neck to help close the leak by external pressure.   **Surgical Management:**  If conservative measures fail after 5-7 days or if the leak is high-volume (>500 mL/day), surgical intervention may be required.   1. **Direct Ligation:**    - Surgical exploration to directly identify and ligate the leaking lymphatic vessel. 2. **Fibrin Glue Application:**    - Use of fibrin glue to seal the leaking area. 3. **Thoracic Duct Ligation:**    - In refractory cases, ligation of the thoracic duct in the thorax may be necessary.   **Bibliographic References:**   1. **Schneble EJ, Graham L, Richmon JD.** Management of Chyle Leak after Head and Neck Surgery: A Systematic Review. **Otolaryngol Head Neck Surg.** 2019 Mar;160(3):363-372. doi: 10.1177/0194599818819616. 2. **Nussenbaum B, Liu JH, Sinard RJ.** Systematic management of chyle fistula: the Southwestern experience and review of the literature. **Otolaryngol Head Neck Surg.** 2000 May;122(5):634-639. doi: 10.1067/mhn.2000.104628. 3. **Rudmik L, Smith TL.** Management of chyle leak after head and neck surgery: A systematic review. **Otolaryngol Head Neck Surg.** 2011 Jun;144(6):1000-1005. doi: 10.1177/0194599811405450.   **Additional Notes:**   - Early identification and management are crucial to prevent complications such as malnutrition, immunosuppression, and infection. - Close monitoring of fluid output, electrolyte balance, and nutritional status is essential in patients with chyle leak. - Multidisciplinary collaboration with nutritionists and interventional radiologists can optimize patient outcomes.   If you need further detailed guidance on specific cases or advanced surgical techniques, please let me know. |
|  |  | QAMAI Scores: Reviewer 1: 24; Reviewer 2: 25; Reviewer 3: 24 | | QAMAI Scores: Reviewer 1: 28; Reviewer 2: 25; Reviewer 3: 28 | |
| Clinical scenario | 5 | I am visiting a patient with moderately differentiated squamous cell carcinoma of the right retromolar trigone. The patient has a history of bilateral tibia and fibula fracture, which occurred 10 years ago, and severe venous insufficiency of the lower limbs. The patient has no other comorbidities. The CT scan shows infiltration of the underlying bone and a 4x5 cm lesion centered on the retromolar trigone, extending laterally to the cheek and medially to the soft palate, surpassing the midline. The CT also detected 3 rounded lymph nodes < 4 cm, compatible with secondary nodes, at level IIa and III on the right. No distant metastases were detected. What is the staging of the tumor? What treatment is indicated for the tumor? What is the first-line reconstructive technique for this specific case? Please provide me with the bibliographical references from which you draw your information. | Given the clinical and imaging findings, the patient's tumor can be staged as follows according to the AJCC 8th Edition (American Joint Committee on Cancer Staging Manual):   1. **Primary Tumor (T):**    - The lesion is larger than 4 cm and involves the retromolar trigone, extending to the cheek and soft palate, surpassing the midline.    - This corresponds to T4a (moderately advanced local disease) due to bone invasion. 2. **Regional Lymph Nodes (N):**    - Presence of 3 lymph nodes, each less than 4 cm in size, suggests N2b (multiple ipsilateral lymph nodes, none larger than 6 cm). 3. **Distant Metastasis (M):**    - No distant metastases are detected, which is M0.   **Staging:**   - Combining these findings, the overall staging is **Stage IVA (T4a, N2, M0)**.   **Treatment Plan**  The treatment of choice for a T4aN2M0 squamous cell carcinoma of the oral cavity involves a multimodal approach:   1. **Surgical Resection:**    - Primary tumor resection with negative margins.    - Neck dissection (selective or modified radical neck dissection) to address the involved lymph nodes. 2. **Reconstruction:**    - Considering the extent of the defect after resection, a free flap reconstruction is typically the first-line approach. For this specific case involving the retromolar trigone and adjacent structures, a **fibular free flap** is commonly used due to its reliability and ability to provide both bone and soft tissue. 3. **Adjuvant Therapy:**    - Postoperative radiotherapy or chemoradiotherapy is recommended, especially given the nodal involvement and the advanced stage of the primary tumor.   **Bibliographical References**   - **AJCC Cancer Staging Manual, 8th Edition** for staging criteria. - **National Comprehensive Cancer Network (NCCN) Guidelines** for the treatment of head and neck cancers. - **Principles and Practice of Head and Neck Surgery and Oncology** for surgical and reconstructive techniques. - **Head and Neck Cancer: Multimodality Management** by Jacques Bernier for detailed approaches to adjuvant therapies. | Seeker: I'm a head and neck surgeon with over 15 years of experience, working in a tertiary-level hospital, and I specialize in oncologic surgery  Mission: I need your advice on how to manage a patient I'm treating  AI Role: You are the world's leading expert in oncologic surgery  Register: correct and specialized scientific language. The information must be based on the most recent and solid scientific evidence. I would like you to also provide me with the bibliographical references from which you draw your information  Targeted question: I am visiting a patient with moderately differentiated squamous cell carcinoma of the right retromolar trigone. The patient has a history of bilateral tibia and fibula fracture, which occurred 10 years ago, and severe venous insufficiency of the lower limbs. The patient has no other comorbidities. The CT scan shows infiltration of the underlying bone and a 4x5 cm lesion centered on the retromolar trigone, extending laterally to the cheek and medially to the soft palate, surpassing the midline. The CT also detected 3 rounded lymph nodes < 4 cm, compatible with secondary nodes, at level IIa and III on the right. No distant metastases were detected. What is the staging of the tumor? What treatment is indicated for the tumor? What is the first-line reconstructive technique for this specific case? | **Staging:**  Based on the provided clinical information, the tumor staging according to the AJCC 8th edition (American Joint Committee on Cancer) for oral cavity cancer would be:   - **Primary Tumor (T):** T4a (Tumor invades through cortical bone, into deep [extrinsic] muscle of tongue, maxillary sinus, or skin of the face) - **Regional Lymph Nodes (N):** N2b (Multiple ipsilateral lymph nodes, none more than 6 cm in greatest dimension) - **Distant Metastasis (M):** M0 (No distant metastasis)   Thus, the clinical stage of the tumor is **Stage IVA** (T4a N2b M0).  **Treatment:**  For Stage IVA squamous cell carcinoma of the retromolar trigone, the standard treatment approach is multimodal, typically involving:   1. **Surgical Resection:**    - Wide local excision of the primary tumor with negative margins.    - Mandibulectomy may be necessary due to bone invasion.    - Neck dissection (modified radical or selective neck dissection) to address the involved lymph nodes (levels IIa and III). 2. **Adjuvant Therapy:**    - Postoperative radiation therapy (PORT) is recommended, especially given the bone invasion and lymph node involvement.    - Concurrent chemoradiotherapy might be considered in cases with high-risk features such as extranodal extension (ENE) or positive surgical margins.   **First-line Reconstructive Technique:**  The reconstructive approach should focus on restoring form and function, particularly in the oral cavity where speech and swallowing are critical. For this patient, the following technique is recommended:   1. **Free Flap Reconstruction:**    - **Fibula Free Flap:** Given the need for bony reconstruction due to mandibular involvement. The fibula free flap allows for reconstruction of the bone defect while providing soft tissue coverage, although careful consideration of the patient’s venous insufficiency is necessary.    - **Anterolateral Thigh (ALT) Free Flap:** An alternative if venous insufficiency precludes the use of the fibula flap. The ALT flap provides ample soft tissue and can be harvested with fascia lata for additional structural support. 2. **Considerations for Venous Insufficiency:**    - Thorough preoperative vascular assessment is necessary.    - Intraoperative monitoring of flap perfusion is critical.    - Postoperative management should include strategies to optimize venous return and minimize the risk of thrombosis.   **References:**   1. **Amin MB, Edge SB, Greene FL, et al.** AJCC Cancer Staging Manual. 8th ed. Springer International Publishing; 2017. 2. **Sacco AG, Cohen EEW.** Current treatment options for recurrent or metastatic head and neck squamous cell carcinoma. J Clin Oncol. 2015;33(29):3305-3313. 3. **Pignon JP, Maître A le, Maillard E, Bourhis J; MACH-NC Collaborative Group.** Meta-analysis of chemotherapy in head and neck cancer (MACH-NC): An update on 93 randomised trials and 17,346 patients. Radiother Oncol. 2009;92(1):4-14. 4. **D'Souza J, Vaidhyanath R, Hughes C.** Reconstruction options for segmental mandibular defects in oral cancer patients. Int J Surg Oncol. 2018;2018:7290651. 5. **Fakhry C, Westra WH, Li S, et al. (2008)** Improved survival of patients with human papillomavirus-positive head and neck squamous cell carcinoma in a prospective clinical trial. J Natl Cancer Inst, 100(4), 261-269. |
|  |  | QAMAI Scores: Reviewer 1: 20; Reviewer 2: 20; Reviewer 3: 21 | | QAMAI Scores: Reviewer 1: 27; Reviewer 2: 29; Reviewer 3: 30 | |
| Clinical scenario | 6 | I have a patient with squamous cell carcinoma of the skin in the temporal region. On CT scan, there is evidence of a suspected lymph node metastasis at the homolateral level II. What is the recommended procedure for this patient? Please provide me with the bibliographical references from which you draw your information. | For a patient with squamous cell carcinoma of the skin in the temporal region and suspected lymph node metastasis at the homolateral level II, the recommended approach involves a combination of surgical and adjuvant treatments.  **Recommended Procedure**   1. **Surgical Excision**: The primary treatment for squamous cell carcinoma of the skin is surgical excision. For tumors with suspected lymph node metastasis, the excision should include wide local excision of the primary tumor with appropriate margins. In the case of lymph node involvement at level II, a neck dissection is typically required to remove the affected lymph nodes. 2. **Radiation Therapy**: Postoperative radiation therapy is recommended for patients with high-risk features, such as perineural invasion, lymphovascular invasion, or positive surgical margins. Radiation is particularly important in cases where the primary tumor is large, involves deep tissues, or when there is extensive lymph node involvement. 3. **Chemotherapy**: For patients with extensive lymph node metastasis or when surgical margins are positive and cannot be cleared with additional surgery, chemotherapy may be added to the radiation therapy regimen. This approach helps to control local and regional disease and reduce the risk of further metastasis. 4. **Immunotherapy**: In cases where the cancer is advanced and not amenable to surgery or radiation alone, immunotherapy targeting the PD-1 receptor has shown efficacy. This treatment can be considered for patients with high-risk, advanced squamous cell carcinoma of the skin.   **Multidisciplinary Care**  Management of such cases requires a multidisciplinary approach involving dermatologists, surgical oncologists, radiation oncologists, and medical oncologists to ensure comprehensive care and optimal outcomes.  **References**   1. National Comprehensive Cancer Network (NCCN) Guidelines Insights: Squamous Cell Skin Cancer, Version 1.2022, Journal of the National Comprehensive Cancer Network, Volume 19, Issue 12, 2021. 2. Johns Hopkins Medicine: Squamous Cell Skin Cancer of the Head and Neck, accessed at [Johns Hopkins Medicine](https://www.hopkinsmedicine.org). 3. Healthline: Stage 4 Squamous Cell Carcinoma: Prognosis and Outlook, accessed at [Healthline](https://www.healthline.com). | Seeker: I'm a head and neck surgeon with over 15 years of experience, working in a tertiary-level hospital, and I specialize in oncologic surgery  Mission: I need your advice on how to manage a patient I'm treating.  AI Role: You are the world's leading expert in oncologic surgery  Register: correct and specialized scientific language. The information must be based on the most recent and solid scientific evidence. I would like you to also provide me with the bibliographical references from which you draw your information  Targeted question: I have a patient with squamous cell carcinoma of the skin in the temporal region. On CT scan, there is evidence of a suspected lymph node metastasis at the homolateral level II. What is the recommended procedure for this patient? please Give me the bibliographical references from which you got your information. | For a patient with squamous cell carcinoma (SCC) of the skin in the temporal region with suspected lymph node metastasis at the homolateral level II, the management involves a multidisciplinary approach. Here are the recommended steps based on current guidelines and evidence:  **1. Confirmation of Diagnosis**   - **Fine-Needle Aspiration (FNA) or Core Biopsy of the Lymph Node:**   - To confirm the presence of metastasis, an FNA or core biopsy of the suspicious lymph node should be performed.   **2. Surgical Management**   - **Wide Local Excision (WLE) of the Primary Tumor:**   - The primary tumor should be excised with appropriate margins (typically 1-2 cm depending on tumor size and location). - **Neck Dissection:**   - For confirmed nodal metastasis, a selective neck dissection involving levels I-III (or I-IV) is recommended.   - The specific levels dissected can be tailored based on the location and extent of nodal involvement. Level II dissection is necessary given the CT findings.   **3. Adjuvant Therapy**   - **Radiation Therapy:**   - Postoperative radiation therapy (PORT) is often recommended for high-risk SCC, especially with nodal involvement.   - Indications include large primary tumors, close or positive margins, extracapsular spread, and multiple positive nodes. - **Chemoradiotherapy:**   - Concurrent chemoradiotherapy may be considered in cases with extracapsular spread or multiple high-risk features.   **4. Follow-Up and Monitoring**   - Regular follow-up with clinical examination and imaging as appropriate. - Monitoring for recurrence or new primary tumors, especially in high-risk patients.   **Key References**   1. **NCCN Guidelines (National Comprehensive Cancer Network):** These guidelines provide comprehensive recommendations for the management of cutaneous squamous cell carcinoma, including surgical margins, indications for neck dissection, and adjuvant therapy.    - Link: NCCN Guidelines for Cutaneous Squamous Cell Carcinoma 2. **British Association of Dermatologists (BAD) Guidelines:**    - These guidelines offer detailed protocols for the diagnosis and management of cutaneous SCC, including surgical and non-surgical options.    - Link: BAD Guidelines for Cutaneous Squamous Cell Carcinoma 3. **American Society of Clinical Oncology (ASCO) Guidelines:**    - ASCO provides guidelines specifically addressing the role of adjuvant therapy in high-risk SCC.    - Link: ASCO Guidelines on Cutaneous Squamous Cell Carcinoma 4. **Recent Reviews and Meta-Analyses:**    - For an overview of the latest evidence, recent meta-analyses and systematic reviews in journals such as *JAMA Dermatology* and *The Lancet Oncology* provide high-level evidence on the management of cutaneous SCC with nodal involvement.   By adhering to these guidelines and incorporating the latest evidence-based practices, you can optimize the management of your patient with temporal region SCC and suspected nodal metastasis. |
|  |  | QAMAI Scores: Reviewer 1: 20; Reviewer 2: 25; Reviewer 3: 20 | | QAMAI Scores: Reviewer 1: 25; Reviewer 2: 26; Reviewer 3: 26 | |
| Clinical scenario | 7 | Which treatment would you suggest for the patient thereafter described?  The patient is a 57-year-old male recently diagnosed with squamous cell carcinoma of the left half of the tongue. Tumor staging is cT4aN2bM0. The patient is an active smoker and and a daily alcohol intake of 3 standard units. The patient has no other significant active disease and his medical record reports no previous surgeries, treatments, or significant conditions. The imaging performed for the tumor is as follows. Contrast-enhanced magnetic resonance imaging report is the following (in quotation marks) “A focal alteration with an ulcerated component is observed at the left lingual margin, with an overall maximum diameter of approximately 5 cm (anteroposterior diameter) and an estimated depth of invasion (DOI) of about 1.6 cm. The alteration affects the extrinsic lingual musculature of the involved side, infiltrating the genioglossus muscle, the hyoglossus muscle, and the caudal portion of the styloglossus and palatoglossus muscles. The sublingual space is partially involved. No evidence of bone erosions. Presence of several enlarged lymph nodes at the right level II, the largest with a diameter of about 1 cm. No evidence of extracapsular extension.” Contrast-enhanced computed tomography report is the following (in quotation marks) “NECK: At the level of the left half of the tongue, there is an area of increased contrast uptake (dimensions approximately AP x LL x CC: 45 x 18 x 45 mm) that is the site of a known heteroplastic lesion, ulcerated, with involvement of the ipsilateral hypoglossal and genioglossal muscles, abutting and without a clear cleavage plane with the ipsilateral mylohyoid and geniohyoid muscles, without encroachment into the contralateral half of the tongue nor evidence of bone involvement. Several heterogeneous oval lymphadenopathies, likely secondary, are detected in the left lateral-cervical area, the largest at level II (21x14 mm) and level III (20x10 mm). No densitometric alterations in the parotid or submandibular glands. Preserved patency of the parapharyngeal fat spaces. Micro-calcification at the lower pole of the left thyroid lobe. CHEST: No pulmonary lesions suspicious for metastasis are evident. Some areas of faint ground-glass consolidation in the apical-dorsal portions of the upper lobes, primarily hypothesized as minor inflammatory phenomena, are to be re-evaluated over time. No pleural effusion nor significant pericardial effusion. Several lymphadenopathies with a short axis slightly over 1 cm are detected in the right hilar region (max 14x13 mm) and others with a short axis under 1 cm in the mediastinum (the largest in the right paratracheal area of 13 x 8 mm and in the Barety's lodge of 14 x 9 mm). BONE: No evident focal bony alterations suspicious for secondary involvement in the bone segments included in the cervical-thoracic scan planes.”  Please provide me with the bibliographical references from which you draw your information. | Given the detailed clinical and imaging findings of the 57-year-old male patient with cT4aN2bM0 squamous cell carcinoma of the left half of the tongue, the treatment plan would typically involve a multidisciplinary approach. Here are the steps and considerations for treatment:  **Multidisciplinary Tumor Board Discussion**  A multidisciplinary team, including a head and neck surgeon, radiation oncologist, medical oncologist, radiologist, pathologist, and supportive care specialists, should discuss the case to formulate an optimal treatment plan.  **Primary Treatment Approach**   1. **Surgical Resection:**    - **Objective:** Achieve clear surgical margins and remove all gross disease.    - **Procedure:** Given the extensive local invasion, a composite resection is likely needed, including partial or total glossectomy with neck dissection (radical or modified radical).    - **Reconstruction:** Free flap reconstruction (e.g., radial forearm or anterolateral thigh flap) to restore form and function of the tongue. 2. **Neck Dissection:**    - **Objective:** Remove metastatic lymph nodes and achieve regional control.    - **Procedure:** Bilateral neck dissection, focusing on levels I-V on the left side and levels II-IV on the right side, given the lymphadenopathy.   **Adjuvant Therapy**   1. **Radiation Therapy:**    - **Indications:** High risk of local-regional recurrence due to T4a stage and multiple lymph node involvement.    - **Procedure:** Intensity-modulated radiation therapy (IMRT) to the primary site and neck nodes.    - **Dose:** Typically 60-70 Gy in fractions over 6-7 weeks. 2. **Concurrent Chemotherapy:**    - **Indications:** Indicated for advanced-stage disease (T4a) and N2b nodal status.    - **Regimen:** Cisplatin-based chemoradiation is the standard.    - **Alternative:** If cisplatin is contraindicated, carboplatin or cetuximab can be considered.   **Additional Considerations**   1. **Smoking Cessation:**    - Essential to reduce treatment complications and improve outcomes.    - Referral to a smoking cessation program. 2. **Alcohol Cessation:**    - Necessary to improve treatment tolerance and reduce recurrence risk.    - Referral to counseling and support services. 3. **Nutritional Support:**    - Pre-treatment assessment by a dietitian.    - Consider prophylactic gastrostomy tube placement for nutritional support during treatment. 4. **Follow-Up and Surveillance:**    - Regular follow-up visits to monitor for recurrence, manage complications, and provide supportive care.    - Imaging and physical exams as per NCCN guidelines.   **References**   1. **National Comprehensive Cancer Network (NCCN) Guidelines for Head and Neck Cancers (Version 3.2024):**    - These guidelines provide comprehensive recommendations for the diagnosis, treatment, and follow-up of head and neck cancers, including squamous cell carcinoma of the tongue. 2. **American Society of Clinical Oncology (ASCO) Clinical Practice Guidelines:**    - ASCO guidelines offer evidence-based recommendations for the management of squamous cell carcinoma of the head and neck. 3. **Head and Neck Cancer: A Multidisciplinary Approach by Louis B. Harrison et al.:**    - This textbook provides detailed insights into the multidisciplinary management of head and neck cancers, including surgical techniques, radiation therapy protocols, and chemotherapy regimens. 4. **UpToDate: Management of Squamous Cell Carcinoma of the Head and Neck:**    - UpToDate articles provide a thorough review of the current standards of care for head and neck cancers, including treatment strategies and supportive care measures.   The treatment plan should be personalized based on the patient's overall health, preferences, and specific tumor characteristics. The patient should be informed about the potential benefits, risks, and side effects of each treatment modality. | Seeker: I'm a head and neck surgeon with over 15 years of experience, working in a tertiary-level hospital, and I specialize in oncologic surgery  Mission: I need your advice on how to manage a patient I'm treating.  AI Role: You are the world's leading expert in oncologic surgery  Register: correct and specialized scientific language. The information must be based on the most recent and solid scientific evidence. I would like you to also provide me with the bibliographical references from which you draw your information  Targeted question: Which treatment would you suggest for the patient thereafter described? The patient is a 57-year-old male recently diagnosed with squamous cell carcinoma of the left half of the tongue. Tumor staging is cT4aN2bM0. The patient is an active smoker and and a daily alcohol intake of 3 standard units. The patient has no other significant active disease and his medical record reports no previous surgeries, treatments, or significant conditions. The imaging performed for the tumor is as follows. Contrast-enhanced magnetic resonance imaging report is the following (in quotation marks) “A focal alteration with an ulcerated component is observed at the left lingual margin, with an overall maximum diameter of approximately 5 cm (anteroposterior diameter) and an estimated depth of invasion (DOI) of about 1.6 cm. The alteration affects the extrinsic lingual musculature of the involved side, infiltrating the genioglossus muscle, the hyoglossus muscle, and the caudal portion of the styloglossus and palatoglossus muscles. The sublingual space is partially involved. No evidence of bone erosions. Presence of several enlarged lymph nodes at the right level II, the largest with a diameter of about 1 cm. No evidence of extracapsular extension.” Contrast-enhanced computed tomography report is the following (in quotation marks) “NECK: At the level of the left half of the tongue, there is an area of increased contrast uptake (dimensions approximately AP x LL x CC: 45 x 18 x 45 mm) that is the site of a known heteroplastic lesion, ulcerated, with involvement of the ipsilateral hypoglossal and genioglossal muscles, abutting and without a clear cleavage plane with the ipsilateral mylohyoid and geniohyoid muscles, without encroachment into the contralateral half of the tongue nor evidence of bone involvement. Several heterogeneous oval lymphadenopathies, likely secondary, are detected in the left lateral-cervical area, the largest at level II (21x14 mm) and level III (20x10 mm). No densitometric alterations in the parotid or submandibular glands. Preserved patency of the parapharyngeal fat spaces. Micro-calcification at the lower pole of the left thyroid lobe. CHEST: No pulmonary lesions suspicious for metastasis are evident. Some areas of faint ground-glass consolidation in the apical-dorsal portions of the upper lobes, primarily hypothesized as minor inflammatory phenomena, are to be re-evaluated over time. No pleural effusion nor significant pericardial effusion. Several lymphadenopathies with a short axis slightly over 1 cm are detected in the right hilar region (max 14x13 mm) and others with a short axis under 1 cm in the mediastinum (the largest in the right paratracheal area of 13 x 8 mm and in the Barety's lodge of 14 x 9 mm). BONE: No evident focal bony alterations suspicious for secondary involvement in the bone segments included in the cervical-thoracic scan planes.” | Given the detailed clinical, imaging, and staging information provided for your patient, a comprehensive treatment approach is necessary to manage the locally advanced squamous cell carcinoma of the tongue (cT4aN2bM0) effectively. Here is a structured treatment plan based on current guidelines and evidence:  **Multidisciplinary Approach**  This case should be managed with a multidisciplinary team including head and neck surgeons, radiation oncologists, medical oncologists, radiologists, pathologists, and supportive care teams.  **Surgical Treatment**  **Primary Tumor Resection:**   - **Procedure:** The patient should undergo a wide local excision of the primary tumor with negative margins. Given the tumor's invasion into the extrinsic tongue muscles and sublingual space, a partial glossectomy with possible composite resection (including resection of involved muscles) is indicated. - **Reconstruction:** Free flap reconstruction (e.g., radial forearm free flap or anterolateral thigh flap) may be necessary for functional and aesthetic rehabilitation.   **Neck Dissection:**   - **Procedure:** A bilateral neck dissection is recommended due to the presence of multiple enlarged lymph nodes, particularly on the left side (levels II and III).   - Left side: Comprehensive neck dissection (levels I-V).   - Right side: Selective neck dissection (levels I-IV) given the cN2b status.   **Adjuvant Therapy**  **Radiation Therapy:**   - **Indication:** Postoperative radiation therapy is indicated due to the advanced stage of the primary tumor (T4a), lymph node involvement (N2b), and high risk of recurrence. - **Regimen:** Intensity-modulated radiation therapy (IMRT) is preferred to minimize toxicity. Standard dose is typically 60-66 Gy in 30-33 fractions to the primary site and nodal basins.   **Chemoradiotherapy:**   - **Indication:** Concurrent chemoradiotherapy is recommended for high-risk features, including T4a tumors and multiple positive lymph nodes. - **Chemotherapy Regimen:** Cisplatin is commonly used at 100 mg/m² every three weeks during radiation therapy, or a weekly regimen at 40 mg/m².   **Lifestyle Modifications**   - **Smoking Cessation:** Essential to improve treatment outcomes and reduce the risk of secondary malignancies. Referral to a smoking cessation program is recommended. - **Alcohol Reduction:** Advising the patient to significantly reduce or cease alcohol intake is important for improving overall prognosis and reducing recurrence risk.   **Follow-Up and Surveillance**   - **Schedule:** Regular follow-up visits for the first two years post-treatment every 1-3 months, then every 4-6 months for the next three years, and annually thereafter. - **Imaging:** Routine surveillance imaging (e.g., CT, MRI) and clinical examinations to monitor for recurrence or metastasis. - **Functional Assessment:** Regular assessment of speech and swallowing function, nutritional status, and psychological support.   **References**   1. National Comprehensive Cancer Network. NCCN Clinical Practice Guidelines in Oncology: Head and Neck Cancers. Version 3.2023. 2. Haddad, R. I., & Shin, D. M. (2008). Recent advances in head and neck cancer. New England Journal of Medicine, 359(11), 1143-1154. 3. Licitra, L., Felip, E., & ESMO Guidelines Working Group. (2011). Squamous cell carcinoma of the head and neck: ESMO Clinical Practice Guidelines for diagnosis, treatment and follow-up. Annals of Oncology, 22(Supplement_6), vi83-vi85. 4. Forastiere, A. A., Zhang, Q., Weber, R. S., Maor, M. H., Goepfert, H., Pajak, T. F., ... & Cooper, J. S. (2013). Long-term results of RTOG 91-11: a comparison of three nonsurgical treatment strategies to preserve the larynx in patients with locally advanced larynx cancer. Journal of Clinical Oncology, 31(7), 845-852. 5. Ang, K. K., Harris, J., Wheeler, R., Weber, R., Rosenthal, D. I., Nguyen-Tan, P. F., ... & Gillison, M. L. (2010). Human papillomavirus and survival of patients with oropharyngeal cancer. New England Journal of Medicine, 363(1), 24-35.   This structured approach aims to provide a comprehensive treatment strategy, considering the aggressive nature of the tumor and the need for multimodal therapy to optimize outcomes. |
|  |  | QAMAI Scores: Reviewer 1: 25; Reviewer 2: 25; Reviewer 3: 26 | | QAMAI Scores: Reviewer 1: 28; Reviewer 2: 29; Reviewer 3: 30 | |
| Clinical scenario | 8 | I'm seeing a patient who has had, for about 4 months, a new growth on the left cheek, just in front of the ear, which is asymptomatic and has undergone slight enlargement over time. For about 10 days, he has had difficulty closing the left eye and raising the eyebrow. What could be the diagnosis? How should I manage this patient? Please provide me with the bibliographical references from which you draw your information. | Based on the patient's symptoms and history, the differential diagnosis could include:   1. **Parotid Gland Tumor (including malignant types like Mucoepidermoid carcinoma or Adenoid cystic carcinoma)**: Parotid tumors can present as a growth near the ear and might affect the facial nerve, leading to facial weakness or paralysis, as seen in this patient. 2. **Facial Nerve Schwannoma**: A growth affecting the facial nerve can cause similar symptoms of facial nerve dysfunction. 3. **Basal Cell Carcinoma**: Though less likely to cause facial nerve palsy, an invasive lesion near the facial nerve might exert pressure or infiltrate the nerve. 4. **Squamous Cell Carcinoma**: This can also present as a progressively enlarging lesion and might affect adjacent structures including nerves. 5. **Benign Lesions (e.g., Pleomorphic Adenoma)**: While benign, these can still exert pressure on the facial nerve causing dysfunction.   **Recommended Management:**   1. **Detailed Clinical Examination**: Assess the lesion for size, consistency, fixation to underlying structures, and involvement of lymph nodes. 2. **Imaging Studies**:    - **Ultrasound**: Initial assessment of the lesion.    - **MRI/CT Scan**: Detailed imaging to assess the extent of the lesion and its effect on surrounding structures, especially the facial nerve. 3. **Biopsy**:    - **Fine Needle Aspiration Cytology (FNAC)**: To determine the nature of the lesion.    - **Incisional Biopsy**: If FNAC is inconclusive or if a more definitive diagnosis is needed. 4. **Referral to a Specialist**:    - **ENT Specialist or Head and Neck Surgeon**: For further assessment and possible surgical intervention.    - **Oncologist**: If malignancy is suspected.   **References:**   1. **Parotid Gland Tumors**:    - Cohen, J. I. (2009). Salivary gland tumors: general considerations. In Salivary Gland Disorders (pp. 33-41). Springer, Berlin, Heidelberg.    - Eveson, J. W., Auclair, P. L., Gnepp, D. R., & El-Naggar, A. K. (2005). Tumours of the salivary glands. Pathology and Genetics of Head and Neck Tumours, 209-282. 2. **Facial Nerve Schwannoma**:    - Saito, D. M., Weiss, A. D., & Bhuta, S. (2007). Facial nerve schwannoma: case report and review of the literature. Otology & Neurotology, 28(5), 800-802.    - Sinha, S., & Mizrachi, A. (2016). Management of facial nerve schwannomas: a review. Journal of Neurological Surgery Part B: Skull Base, 77(03), 229-235. 3. **Basal Cell Carcinoma**:    - Cameron, M. C., Lee, E., Hibler, B. P., Barker, C. A., Mori, S., Cordova, M., & Rossi, A. M. (2019). Basal cell carcinoma: Epidemiology; pathophysiology; clinical and histologic subtypes; and disease associations. Journal of the American Academy of Dermatology, 80(2), 303-317.    - Lear, J. T., & Smith, A. G. (1997). Basal cell carcinoma. Postgraduate Medical Journal, 73(864), 538-542. 4. **Squamous Cell Carcinoma**:    - Alam, M., & Ratner, D. (2001). Cutaneous squamous-cell carcinoma. New England Journal of Medicine, 344(13), 975-983.    - Burton, K. A., Ashack, K. A., & Khachemoune, A. (2016). Cutaneous squamous cell carcinoma: a review of high-risk and metastatic disease. American Journal of Clinical Dermatology, 17, 491-508. 5. **Pleomorphic Adenoma**:    - Gnepp, D. R. (1991). Malignant mixed tumors of the salivary glands: a review. Pathology Annual, 26, 247-277.    - Vander Poorten, V. L., Balm, A. J., Hilgers, F. J., & Keus, R. B. (1999). Management of parotid pleomorphic adenoma. Acta Oto-Laryngologica, 119(6), 625-631.   For a more precise diagnosis and management plan, a thorough examination and appropriate imaging and biopsy are crucial. Referral to specialists in ENT or oncology will also be essential in guiding treatment. | Seeker: I'm a head and neck surgeon with over 15 years of experience, working in a tertiary-level hospital, and I specialize in oncologic surgery  Mission: I need your advice on how to manage a patient I'm treating.  AI Role: You are the world's leading expert in oncologic surgery.  Register: correct and specialized scientific language. The information must be based on the most recent and solid scientific evidence. I would like you to also provide me with the bibliographical references from which you draw your information  Targeted question: I'm seeing a patient who has had, for about 4 months, a new growth on the left cheek, just in front of the ear, which is asymptomatic and has undergone slight enlargement over time. For about 10 days, he has had difficulty closing the left eye and raising the eyebrow. What could be the diagnosis? How should I manage this patient? | Based on the clinical presentation of a new growth on the left cheek, just in front of the ear, which is asymptomatic and has enlarged slightly over time, combined with recent onset of difficulty closing the left eye and raising the eyebrow, the differential diagnosis should include:   1. **Parotid Gland Neoplasm**:    - The location of the growth suggests involvement of the parotid gland. The facial nerve (cranial nerve VII) passes through the parotid gland, and its involvement can lead to facial weakness or paralysis.    - **Benign Tumors**: Pleomorphic adenoma is the most common benign tumor of the parotid gland. Warthin tumor is another possibility.    - **Malignant Tumors**: Mucoepidermoid carcinoma and adenoid cystic carcinoma are the most common malignant tumors. These tumors can invade local structures, including the facial nerve. 2. **Facial Nerve Palsy**:    - The recent onset of difficulty in closing the left eye and raising the eyebrow is indicative of facial nerve involvement, possibly due to compression or invasion by the growth.   **Diagnostic Workup**   1. **Imaging Studies**:    - **Ultrasound**: Initial evaluation to assess the nature of the mass (solid vs cystic) and its relation to the parotid gland.    - **MRI**: Preferred imaging modality to evaluate the extent of the lesion, its relationship with surrounding structures, and to assess facial nerve involvement.    - **CT Scan**: Useful if there is suspicion of bony involvement or to evaluate regional lymph nodes. 2. **Fine-Needle Aspiration Biopsy (FNAB)**:    - To obtain a tissue diagnosis. FNAB can help differentiate between benign and malignant lesions and guide further management.   **Management**   1. **Surgical Intervention**:    - **Benign Tumors**: Superficial or total parotidectomy with preservation of the facial nerve, if possible.    - **Malignant Tumors**: More extensive surgery, possibly including total parotidectomy and neck dissection, depending on the stage and extent of the tumor. Facial nerve reconstruction may be required if the nerve is involved. 2. **Adjuvant Therapy**:    - **Radiotherapy**: May be indicated in malignant cases, especially if there is perineural invasion, positive margins, or high-grade histology.    - **Chemotherapy**: Considered in advanced or metastatic disease. 3. **Multidisciplinary Approach**:    - Collaboration with a radiologist, pathologist, oncologist, and possibly a reconstructive surgeon is essential for optimal management of the patient.   **Prognosis**   - The prognosis depends on the histological type of the tumor, stage at diagnosis, and the extent of facial nerve involvement. Benign tumors generally have an excellent prognosis post-surgery. Malignant tumors require close follow-up and may have a variable prognosis based on the aforementioned factors.   **References**   1. **Bradley PJ, McGurk M.** Incidence of salivary gland neoplasms. **Otolaryngol Head Neck Surg.** 2013;149(1):122-129. 2. **Ellis GL, Auclair PL.** Tumors of the Salivary Glands. **AFIP Atlas of Tumor Pathology.** Series 4. Washington, DC: Armed Forces Institute of Pathology; 2008. 3. **Spiro RH.** Salivary neoplasms: overview of a 35-year experience with 2,807 patients. **Head Neck Surg.** 1986;8(3):177-184. 4. **Seethala RR, Stenman G.** Update from the 4th Edition of the World Health Organization Classification of Head and Neck Tumours: Tumors of the Salivary Gland. **Head Neck Pathol.** 2017;11(1):55-67.   Please ensure that you conduct a thorough clinical evaluation and utilize the appropriate diagnostic tools to confirm the diagnosis and tailor the treatment plan accordingly. |
|  |  | QAMAI Scores: Reviewer 1: 25; Reviewer 2: 25; Reviewer 3: 24 | | QAMAI Scores: Reviewer 1: 26; Reviewer 2: 23; Reviewer 3: 29 | |
| Theoretical question | 1 | Can you describe step by step the procedure for a modified radical neck dissection type III? Please provide me with the bibliographical references from which you draw your information. | The modified radical neck dissection type III, also known as selective neck dissection, is a surgical procedure to remove lymph nodes from the neck that may contain cancer cells. This surgery aims to preserve certain structures in the neck to reduce morbidity. Here is a detailed step-by-step description of the procedure:  **Preoperative Preparation**   1. **Preoperative Assessment**: The patient is evaluated with imaging studies like CT or MRI to determine the extent of the disease. 2. **Informed Consent**: The surgeon explains the procedure, risks, and benefits to the patient, and obtains informed consent.   **Surgical Procedure**   1. **Anesthesia**: The patient is placed under general anesthesia. 2. **Positioning**: The patient is positioned supine with the head turned to the opposite side of the surgery. The neck is extended slightly. 3. **Incision**: A curvilinear incision is made along a natural skin crease, extending from the mastoid process behind the ear to the midline of the neck, just above the clavicle. 4. **Flap Elevation**: Subplatysmal flaps are elevated to expose the underlying structures of the neck. 5. **Identification of Landmarks**: Important anatomical landmarks such as the sternocleidomastoid muscle, internal jugular vein, and the spinal accessory nerve are identified. 6. **Dissection**:    - **Lymph Node Levels I-III**: The dissection begins with the removal of lymph nodes from levels I to III (submental, submandibular, and upper jugular groups).    - **Preservation of Structures**: In modified radical neck dissection type III, specific structures such as the spinal accessory nerve, internal jugular vein, and sternocleidomastoid muscle are preserved.    - **Spinal Accessory Nerve**: The nerve is carefully dissected free from surrounding tissue to avoid damage.    - **Internal Jugular Vein and Sternocleidomastoid Muscle**: These structures are preserved, and lymphatic tissue around them is carefully removed. 7. **Hemostasis**: Bleeding vessels are coagulated or ligated to maintain a clear surgical field. 8. **Wound Closure**: The wound is closed in layers. Drains are often placed to prevent fluid accumulation. 9. **Dressing**: Sterile dressings are applied to the surgical site.   **Postoperative Care**   1. **Monitoring**: The patient is monitored for signs of complications such as bleeding, infection, or nerve damage. 2. **Drain Management**: Drains are monitored and typically removed a few days after surgery once drainage decreases. 3. **Rehabilitation**: Physical therapy may be recommended to help with shoulder function if the spinal accessory nerve was manipulated.   **Follow-Up**   1. **Pathology Report**: The excised tissue is sent to pathology for examination to determine the presence and extent of cancer cells. 2. **Further Treatment**: Depending on the pathology results, additional treatments such as radiation or chemotherapy may be recommended.   The modified radical neck dissection type III is performed with the goal of removing cancerous lymph nodes while minimizing postoperative complications by preserving vital neck structures​ ([Medicine LibreTexts](https://med.libretexts.org/Bookshelves/Anatomy_and_Physiology/Atlas_of_Otolaryngology_Head_and_Neck_Operative_Surgery/01%3A_Head_and_Neck/1.20%3A_Neck_Dissection-_Modified_and_Radical))​​ ([AHNS](https://www.ahns.info/neck-dissection/))​​ ([Memorial Sloan Kettering Cancer Center](https://www.mskcc.org/cancer-care/patient-education/neck-dissection))​. | Seeker: I am a fifth-year resident specializing in head and neck surgery.  Mission: I need your help to prepare for my final residency exam.  AI Role: You are a full professor of head and neck surgery at the most prestigious university in the world.  Register: correct and specialized scientific language. The information must be based on the most recent and solid scientific evidence. I would like you to also provide me with the bibliographical references from which you draw your information  Targeted question: Can you describe step by step the procedure for a modified radical neck dissection type III? | Certainly. A Modified Radical Neck Dissection Type III (MRND Type III) is a surgical procedure commonly performed for the management of head and neck malignancies. It involves the removal of lymph node groups I-V, preserving the spinal accessory nerve, the internal jugular vein, and the sternocleidomastoid muscle. Here is a step-by-step description of the procedure:  **Preoperative Preparation**   1. **Patient Positioning:** The patient is placed in a supine position with the head turned away from the side of the dissection. A shoulder roll may be used to extend the neck. 2. **Anesthesia:** General anesthesia is administered. 3. **Skin Preparation and Draping:** The neck is prepared and draped in a sterile manner.   **Incision and Flap Elevation**   1. **Incision:** A modified Schobinger or apron incision is made, extending from the mastoid process down to the midline of the neck, curving around the clavicle. 2. **Subplatysmal Flap Elevation:** Subplatysmal flaps are elevated superiorly to the inferior border of the mandible and inferiorly to the clavicle. Care is taken to preserve the platysma muscle to protect the marginal mandibular branch of the facial nerve.   **Dissection of Lymph Node Levels**   1. **Level I Dissection:** The dissection begins at Level I, where the submandibular gland is removed, and the lymph nodes in the submental and submandibular triangle are dissected. The marginal mandibular nerve is preserved. 2. **Level II Dissection:** The sternocleidomastoid muscle (SCM) is retracted, and the spinal accessory nerve is identified and preserved. The dissection proceeds along the internal jugular vein, which is preserved, removing lymph nodes along the carotid sheath. 3. **Level III Dissection:** The dissection continues inferiorly along the carotid sheath, preserving the internal jugular vein and spinal accessory nerve. The lymph nodes are removed en bloc. 4. **Level IV Dissection:** The dissection extends inferiorly to the clavicle, removing the lower jugular lymph nodes. The phrenic nerve and brachial plexus are identified and preserved. 5. **Level V Dissection:** The posterior triangle lymph nodes are dissected, taking care to preserve the spinal accessory nerve and avoid injury to the brachial plexus and phrenic nerve.   **Hemostasis and Closure**   1. **Hemostasis:** Careful hemostasis is achieved throughout the procedure using ligatures and electrocautery. 2. **Drain Placement:** One or two closed suction drains are placed in the neck to prevent hematoma formation. 3. **Wound Closure:** The wound is closed in layers. The platysma is reapproximated, followed by subcutaneous tissue and skin closure, usually with interrupted sutures.   **Postoperative Care**   1. **Monitoring:** The patient is monitored postoperatively for complications such as hematoma, seroma, infection, and nerve injuries. 2. **Drain Management:** Drains are typically removed once the output decreases to an acceptable level, usually within a few days. 3. **Follow-Up:** Regular follow-up is essential to monitor for recurrence and manage any postoperative complications.   **References**   1. Robbins KT. Classification of neck dissections: current concepts and future considerations. Otolaryngol Clin North Am. 1998;31:639-655. 2. Medina JE. A rational classification of neck dissections. Otolaryngol Head Neck Surg. 1989;100(3):169-176. 3. Sheahan P. Management of neck metastases in head and neck cancer. J Surg Oncol. 2008;97(8):644-649. 4. Robbins KT, Medina JE, Wolfe GT, et al. Standardizing neck dissection terminology. Arch Otolaryngol Head Neck Surg. 1991;117(6):601-605.   These steps provide a detailed overview of a Modified Radical Neck Dissection Type III. However, each case may require specific adjustments based on the patient's anatomy and tumor characteristics. |
|  |  | QAMAI Scores: Reviewer 1: 16; Reviewer 2: 16; Reviewer 3: 20 | | QAMAI Scores: Reviewer 1: 23; Reviewer 2: 21; Reviewer 3: 24 | |
| Theoretical question | 2 | What are the therapeutic options for Frey's Syndrome? Please provide me with the bibliographical references from which you draw your information. | Frey's Syndrome, also known as gustatory sweating, can be managed through various therapeutic options. The main treatments include:   1. **Topical Antiperspirants and Anticholinergics**:    - **Aluminum Chloride**: This is often used first to block sweat ducts. Anticholinergic ointments like scopolamine or glycopyrrolate can also be applied topically to reduce sweating​ ([Cleveland Clinic](https://my.clevelandclinic.org/health/diseases/25185-freys-syndrome))​​ ([Salivary Gland Surgery LA](https://parotid.net/freys-syndrome-treatment/))​. 2. **Botulinum Toxin Injections**:    - **Botox (Botulinum Toxin Type A)**: Injections of botulinum toxin are considered highly effective, providing relief for several months by blocking nerve signals to sweat glands. This method has shown a success rate of around 98% and is often preferred due to its efficacy and safety​ ([National Organization for Rare Disorders](https://rarediseases.org/rare-diseases/frey-syndrome/))​​ ([Verywell Health](https://www.verywellhealth.com/frey-syndrome-sweating-after-eating-4145385))​. 3. **Surgical Interventions**:    - **Tympanic Neurectomy**: Cutting the tympanic nerve to stop the nerve signals causing sweating.    - **Interposition of Tissue Grafts**: Surgical placement of tissue such as fascia or muscle between the skin and the parotid bed to prevent nerve regeneration and sweating. This method can also address cosmetic concerns following parotid surgery​ ([Cureus](https://www.cureus.com/articles/77968-freys-syndrome-a-review-of-aetiology-and-treatment))​​ ([Salivary Gland Surgery LA](https://parotid.net/freys-syndrome-treatment/))​. 4. **Radiotherapy**:    - Although less commonly used due to potential long-term side effects, low-dose radiotherapy can be an option to reduce the activity of sweat glands​ ([Cleveland Clinic](https://my.clevelandclinic.org/health/diseases/25185-freys-syndrome))​. 5. **Lifestyle and Behavioral Changes**:    - Avoiding foods that trigger a strong salivary response, such as spicy, sour, or salty foods, can help manage symptoms in some individuals​ ([Cleveland Clinic](https://my.clevelandclinic.org/health/diseases/25185-freys-syndrome))​​ ([National Organization for Rare Disorders](https://rarediseases.org/rare-diseases/frey-syndrome/))​. 6. **Preventive Measures During Initial Surgery**:    - Using surgical techniques that minimize nerve damage, such as flap reconstruction, can help prevent the onset of Frey's Syndrome following parotid gland surgery​ ([Salivary Gland Surgery LA](https://parotid.net/freys-syndrome-treatment/))​.   These treatments can be tailored to the severity of the symptoms and the preferences of the patient. Consulting with a healthcare provider is essential to determine the most appropriate management plan. For more detailed information on Frey's Syndrome and its treatments, you can visit resources like the Cleveland Clinic, NORD, Verywell Health, Cureus, and Parotid.net​ ([Cleveland Clinic](https://my.clevelandclinic.org/health/diseases/25185-freys-syndrome))​​ ([National Organization for Rare Disorders](https://rarediseases.org/rare-diseases/frey-syndrome/))​​ ([Verywell Health](https://www.verywellhealth.com/frey-syndrome-sweating-after-eating-4145385))​​ ([Cureus](https://www.cureus.com/articles/77968-freys-syndrome-a-review-of-aetiology-and-treatment))​​ ([Salivary Gland Surgery LA](https://parotid.net/freys-syndrome-treatment/))​. | Seeker: I am a fifth-year resident specializing in head and neck surgery.  Mission: I need help managing a patient  AI Role: You are a full professor of head and neck surgery at the most prestigious university in the world.  Register: correct and specialized scientific language. The information must be based on the most recent and solid scientific evidence. I would like you to also provide me with the bibliographical references from which you draw your information  Targeted question: What are the therapeutic options for Frey's Syndrome? | **Frey's Syndrome**, also known as gustatory sweating, typically manifests as sweating and flushing in the preauricular area during meals. This condition usually arises following parotidectomy or other surgical interventions near the parotid gland, due to aberrant reinnervation of sweat glands by parasympathetic fibers originally meant for the parotid salivary glands.  **1. Topical Treatments**  **1.1. Anticholinergic Agents:**   - **Glycopyrrolate:** Applied as a 2% cream, glycopyrrolate has been shown to be effective in reducing gustatory sweating. - **Scopolamine:** Another anticholinergic agent that can be used in the form of a transdermal patch.   **1.2. Aluminum Chloride:**   - Often used for hyperhidrosis, 20% aluminum chloride hexahydrate can be effective in reducing sweating when applied topically.   **2. Botulinum Toxin (Botox) Injections**  Botulinum toxin type A (e.g., Botox, Dysport) injections have become the first-line treatment for Frey's Syndrome due to their effectiveness and safety profile. The toxin works by inhibiting acetylcholine release at the neuroglandular junction, thereby reducing sweating. The typical dosage ranges from 50 to 100 units, injected subcutaneously into the affected area.  **Efficacy:**   - Studies have demonstrated significant improvement in symptoms, with effects lasting between 6 to 18 months before requiring repeat injections.   **3. Surgical Interventions**  **3.1. Tympanic Neurectomy:**   - This procedure involves the surgical resection of the tympanic nerve, which is a branch of the glossopharyngeal nerve responsible for parasympathetic innervation to the parotid gland. Although effective, it carries the risk of complications such as taste disturbances and xerostomia.   **3.2. Interpositional Grafting:**   - Interpositional grafts, such as temporoparietal fascia or muscle flaps, can be placed between the skin and the parotid bed during parotidectomy to prevent reinnervation of sweat glands.   **3.3. Subcutaneous Surgical Options:**   - Subcutaneous tissue rearrangement or adipofascial flaps can be utilized to create a barrier between the skin and the underlying parotid gland, thereby reducing the aberrant reinnervation.   **4. Radiotherapy**  Low-dose radiotherapy has been used in some cases to reduce the incidence of Frey's Syndrome, though it is less commonly employed due to potential adverse effects and the availability of less invasive treatments.  **5. Other Pharmacological Options**  **5.1. Oral Anticholinergics:**   - Medications such as oral glycopyrrolate can be prescribed, although systemic side effects may limit their use.   **5.2. Antihistamines:**   - Some patients have found relief with antihistamines, though evidence supporting their efficacy is limited.   **References**   1. **Li C, Wu F, Zhang Q, Gao Q, Shi Z, Li L. Interventions for the treatment of Frey's syndrome. *Cochrane Database Syst Rev*. 2015;2015(3):CD009959.** 2. **Motz KM, Kim YJ. Auriculotemporal Syndrome (Frey Syndrome). *Otolaryngol Clin North Am*. 2016;49(2):501-509.** 3. **Blanc S, Bourrier T, Boralevi F, et al. Frey Syndrome. *J Pediatr*. 2016;174:211-217.e2.**   These references provide a robust basis for the current therapeutic approaches to managing Frey's Syndrome, reflecting the most recent and high-quality scientific evidence available. |
|  |  | QAMAI Scores: Reviewer 1: 22; Reviewer 2: 23; Reviewer 3: 22 | | QAMAI Scores: Reviewer 1: 24; Reviewer 2: 25; Reviewer 3: 28 | |
| Theoretical question | 3 | What are the serum markers that have shown some reliability in predicting the risk of occult lymph node metastasis in oral squamous cell carcinomas? Please provide me with the bibliographical references from which you draw your information. | Several serum markers have shown promise in predicting the risk of occult lymph node metastasis in oral squamous cell carcinoma (OSCC). Notable markers include:   1. **Cytokeratin 19 fragments (CYFRA 21-1)**: Elevated levels have been associated with a higher risk of lymph node metastasis. 2. **Matrix metalloproteinases (MMPs)**: Particularly MMP-2 and MMP-9, which are involved in tumor invasion and metastasis. 3. **Eosinophils**: Increased eosinophil count has been linked to occult lymph node metastasis. 4. **Interleukin-6 (IL-6)**: Higher serum IL-6 levels can indicate increased metastatic potential.   These markers can assist in the early identification of patients at higher risk, potentially improving treatment outcomes​ ([Karger](https://karger.com/orl/article/86/1/32/869871/Blood-Markers-Predicting-Clinically-Occult-Lymph#:~:text=URL%3A%20https%3A%2F%2Fkarger.com%2Forl%2Farticle%2F86%2F1%2F32%2F869871%2FBlood))​. | Seeker: I am a fifth-year resident specializing in head and neck surgery.  Mission: I need your help to prepare for my final residency exam.  AI Role: You are a full professor of head and neck surgery at the most prestigious university in the world.  Register: correct and specialized scientific language. The information must be based on the most recent and solid scientific evidence. I would like you to also provide me with the bibliographical references from which you draw your information  Targeted question: What are the serum markers that have shown some reliability in predicting the risk of occult lymph node metastasis in oral squamous cell carcinomas? | Recent advancements in molecular oncology have identified several serum markers that exhibit potential in predicting the risk of occult lymph node metastasis (LNM) in patients with oral squamous cell carcinoma (OSCC). These markers can be crucial in tailoring personalized treatment strategies, particularly in determining the need for elective neck dissection. Here are some of the most studied and promising serum markers:   1. **Cytokeratin Fragments (CYFRA 21-1)**    - **Significance**: Elevated levels of CYFRA 21-1 have been correlated with a higher risk of lymph node metastasis in OSCC. It reflects the presence of tumor cells with epithelial origin undergoing necrosis or apoptosis.    - **Reference**: Zhuang SM, Zhang YS, Tan Y, Liu Z, Xu YM. CYFRA 21-1 levels in the serum of patients with oral squamous cell carcinoma. **Oral Oncol**. 2015;51(6):588-593. doi:10.1016/j.oraloncology.2015.03.006. 2. **Squamous Cell Carcinoma Antigen (SCCA)**    - **Significance**: SCCA is a tumor-associated antigen found in the serum of patients with squamous cell carcinomas. Its elevated levels have been linked to a greater likelihood of metastasis and poorer prognosis.    - **Reference**: Duk JM, Groenier KH, de Bruijn HW, et al. Pretreatment serum squamous cell carcinoma antigen: a newly identified prognostic factor in early-stage cervical carcinoma. **J Clin Oncol.** 1996;14(1):111-118. doi:10.1200/JCO.1996.14.1.111 3. **Matrix Metalloproteinases (MMPs)**    - **Significance**: Particularly MMP-2 and MMP-9, these enzymes play a role in extracellular matrix degradation and are often upregulated in cancer. Their serum levels have been associated with increased invasive potential and lymph node metastasis.    - **Reference**: Guttman D, Kessler A, Pitaro K, Ben-Izhak O, Yihai R, Vaiman M. Serum levels of matrix metalloproteinases as a marker for lymph node metastasis in patients with oral squamous cell carcinoma. **Oral Oncol**. 2004;40(7):698-703. doi:10.1016/j.oraloncology.2004.02.004. 4. **Tissue Polypeptide-Specific Antigen (TPS)**    - **Significance**: TPS is a marker of cell proliferation, and higher serum levels have been linked to aggressive tumor behavior and metastatic potential in OSCC.    - **Reference**: Chen P, Jiao L, Ren F, Wang DB. Clinical value of serum squamous cell carcinoma antigen levels in predicting chemosensitivity, lymph node metastasis, and prognosis in patients with cervical squamous cell carcinoma. **BMC Cancer.** 2020 May 14;20(1):423. doi: 10.1186/s12885-020-06934-x. 5. **Interleukin-6 (IL-6)**    - **Significance**: IL-6 is a pro-inflammatory cytokine involved in cancer progression and metastasis. Elevated serum IL-6 levels have been associated with poor prognosis and increased likelihood of LNM.    - **Reference**: Chen P, Jiao L, Ren F, Wang DB. Clinical value of serum squamous cell carcinoma antigen levels in predicting chemosensitivity, lymph node metastasis, and prognosis in patients with cervical squamous cell carcinoma. **BMC Cancer.** 2020 May 14;20(1):423. doi: 10.1186/s12885-020-06934-x. 6. **Vascular Endothelial Growth Factor (VEGF)**    - **Significance**: VEGF plays a critical role in angiogenesis and lymphangiogenesis, processes that facilitate tumor metastasis. Increased serum VEGF levels have been linked to higher metastatic rates in OSCC.    - **Reference**: Higashi Y, Nakamura K, Takaoka R, Tani M, Noma Y, Mori K, Yamashiro K, Yokoyama S, Hamada T, Sugiura T. Identification of Neck Lymph Node Metastasis-Specific microRNA-Implication for Use in Monitoring or Prediction of Neck Lymph Node Metastasis. **Cancers (Basel).** 2023 Jul 25;15(15):3769. doi: 10.3390/cancers15153769. 7. **Epidermal Growth Factor Receptor (EGFR)**    - **Significance**: EGFR overexpression and high serum levels are associated with aggressive tumor behavior and increased risk of LNM in OSCC.    - **Reference**: Su NW, Leu YS, Lee JC, et al. EGF and EGFR genetic polymorphisms predict prognosis in locally advanced pharyngolaryngeal squamous cell carcinoma patients receiving postoperative concurrent chemoradiotherapy. **Onco Targets Ther**. 2014;7:2197-2204. Published 2014 Nov 28. doi:10.2147/OTT.S70188   While these serum markers show promise, their clinical utility often requires further validation in larger, multi-center studies. It is also important to consider that combining multiple biomarkers may enhance predictive accuracy compared to using a single marker. Integrating these serum markers with clinical and imaging findings could provide a more comprehensive assessment of metastatic risk in OSCC patients. |
|  |  | QAMAI Scores: Reviewer 1: 15; Reviewer 2: 17; Reviewer 3: 18 | | QAMAI Scores: Reviewer 1: 26; Reviewer 2: 30; Reviewer 3: 30 | |
| Theoretical question | 4 | What is the first-line treatment in an asymptomatic patient with an incidental finding of a 1 cm stone in the hilum of the submandibular gland? Please provide me with the bibliographical references from which you draw your information. | The first-line treatment for an asymptomatic patient with an incidental finding of a 1 cm stone in the hilum of the submandibular gland typically involves conservative management. This approach includes measures such as:   1. **Hydration**: Encouraging the patient to drink plenty of fluids to help increase saliva flow and potentially dislodge the stone. 2. **Sialogogues**: Using substances that stimulate saliva production, like lemon drops or sour candies, to help expel the stone. 3. **Massage and Heat**: Applying gentle massage and warm compresses to the affected area to promote salivary flow and stone movement.   If conservative methods do not lead to resolution, minimally invasive procedures such as sialendoscopy can be considered. Sialendoscopy is an endoscopic technique that allows for direct visualization and removal of the stone through the salivary duct. This procedure is typically performed under local or general anesthesia and has a high success rate with minimal complications.  In cases where the stone is too large or difficult to remove via sialendoscopy, surgical options may be explored. These can include transoral stone removal, which involves a small incision in the mouth to access and extract the stone. This method has shown good outcomes in terms of preserving gland function and patient quality of life.  Ultimately, the choice of treatment depends on the size and location of the stone, as well as the presence of any symptoms or complications such as infections​ ([Cleveland Clinic](https://my.clevelandclinic.org/health/diseases/24344-sialolithiasis))​​ ([TeachMeSurgery](https://teachmesurgery.com/ent/neck/sialolithiasis/))​​ ([Penn Medicine](https://www.pennmedicine.org/for-patients-and-visitors/patient-information/conditions-treated-a-to-z/salivary-gland-stone))​.  For more detailed information, you can refer to resources from the Cleveland Clinic, Penn Medicine, and TeachMeSurgery. | Seeker: I am a fifth-year resident specializing in head and neck surgery.  Mission: I need your help to prepare for my final residency exam.  AI Role: You are a full professor of head and neck surgery at the most prestigious university in the world.  Register: correct and specialized scientific language. The information must be based on the most recent and solid scientific evidence. I would like you to also provide me with the bibliographical references from which you draw your information  Targeted question: What is the first-line treatment in an asymptomatic patient with an incidental finding of a 1 cm stone in the hilum of the submandibular gland? | The management of an asymptomatic patient with an incidental finding of a 1 cm stone in the hilum of the submandibular gland requires a careful and balanced approach, considering the potential risks and benefits of intervention versus observation.  **First-Line Treatment**  For an asymptomatic patient, the initial approach typically involves conservative management and observation. Given the stone's location and size, there is a potential risk of future symptoms or complications, but immediate intervention is not always necessary if the patient is asymptomatic.  **Conservative Management**   1. **Hydration**: Encourage the patient to maintain adequate hydration to promote salivary flow, which might help in preventing further stone growth or additional stone formation. 2. **Sialogogues**: Advising the use of sialogogues (substances that stimulate saliva production) such as lemon drops or sour candies may help in increasing salivary flow and potentially dislodging smaller stones. 3. **Massage and Warm Compresses**: Gentle massage of the gland and the use of warm compresses can help in promoting ductal patency and salivary flow. 4. **Regular Follow-up**: Schedule regular follow-up visits to monitor the patient for the development of symptoms or any signs of infection or obstruction.   **Indications for Further Intervention**  Intervention might become necessary if the patient develops symptoms such as pain, swelling, recurrent infections, or obstructive sialadenitis. The following procedures may be considered based on the clinical scenario:   1. **Sialendoscopy**: A minimally invasive procedure that allows direct visualization of the duct system and facilitates stone removal. This technique has gained popularity due to its high success rate and low morbidity. 2. **Transoral Stone Removal**: For stones located near the hilum, this surgical approach may be utilized. It involves accessing the stone via an incision in the floor of the mouth. 3. **Lithotripsy**: Extracorporeal shock wave lithotripsy (ESWL) can be considered to fragment the stone, making it easier to pass or to be removed via sialendoscopy.   **Bibliographical References**   1. **Surgical Treatment**:    - Koch, M., Zenk, J., Iro, H. (2009). Diagnostic and interventional sialendoscopy in obstructive diseases of the salivary glands. Otolaryngologic Clinics of North America, 42(6), 1173-1192.    - Nahlieli, O., Nakar, L. H., Nazarian, Y., Turner, M. D. (2006). Sialendoscopy: a new approach to salivary gland obstructive pathology. Journal of the American Dental Association, 137(10), 1394-1400. 2. **Conservative Management**:    - Escudier, M. P., McGurk, M. (1999). Symptomatic and asymptomatic salivary stones: a retrospective study of treatment outcome. Annals of the Royal College of Surgeons of England, 81(4), 259-262.    - Rauch, S., Gorlin, R. J. (1965). Diseases of the Salivary Glands. Philadelphia: W.B. Saunders.   **Summary**  The first-line treatment for an asymptomatic patient with a 1 cm stone in the hilum of the submandibular gland is conservative management, emphasizing hydration, sialogogues, massage, and regular follow-up. Surgical intervention should be reserved for symptomatic cases or if complications arise. Regular monitoring is crucial to ensure timely intervention if the clinical scenario changes.  4o  Inizio modulo  Fine modulo |
|  |  | QAMAI Scores: Reviewer 1: 18; Reviewer 2: 15; Reviewer 3: 13 | | QAMAI Scores: Reviewer 1: 25; Reviewer 2: 21; Reviewer 3: 24 | |
| Theoretical question | 5 | What are the peculiar clinical characteristics of adenoid cystic carcinomas of the salivary glands that differentiate them from other types of tumors at these sites? Please provide bibliographic references to your sources Please provide me with the bibliographical references from which you draw your information. | Adenoid cystic carcinoma (ACC) of the salivary glands presents several peculiar clinical characteristics that distinguish it from other types of salivary gland tumors.   1. **Histopathological Features**: ACC is characterized by its distinct histopathological patterns, which include tubular, cribriform, and solid structures. The cribriform pattern is often described as having a "Swiss cheese" appearance. The solid subtype is associated with a more aggressive behavior and poorer prognosis compared to the tubular and cribriform subtypes​ ([NCBI](https://www.ncbi.nlm.nih.gov/books/NBK557855/))​​ ([Springer](https://link.springer.com/article/10.1007/s12105-020-01210-7))​. 2. **Growth Pattern**: ACC typically exhibits a slow but relentless growth pattern. Despite its indolent nature, it has a high propensity for perineural invasion, which can lead to significant pain and facial paralysis. This invasion of nerve sheaths is a hallmark of ACC and contributes to its tendency for local recurrence​ ([MDPI](https://www.mdpi.com/2077-0383/13/1/267))​​ ([Springer](https://link.springer.com/article/10.1007/s12105-020-01210-7))​. 3. **Metastatic Potential**: ACC has a notable tendency for distant metastasis, particularly to the lungs, even many years after the initial treatment. This metastatic spread often occurs without widespread lymph node involvement, which is less common in ACC compared to other salivary gland malignancies​ ([IIAR Journals](https://ar.iiarjournals.org/content/37/6/3045))​​ ([MDPI](https://www.mdpi.com/2077-0383/13/1/267))​. 4. **Prognosis and Recurrence**: While the 5-year survival rate for ACC patients is relatively high, around 75%, the 10-year survival rate drops significantly to about 20%. This decline is due to the high rates of late distant metastases and local recurrences. Prognostic factors for poorer outcomes include larger tumor size, presence of perineural invasion, and solid histological pattern​ ([NCBI](https://www.ncbi.nlm.nih.gov/books/NBK557855/))​​ ([IIAR Journals](https://ar.iiarjournals.org/content/37/6/3045))​. 5. **Treatment Challenges**: Surgical resection is the primary treatment modality, often followed by radiation therapy. Complete surgical excision can be challenging due to the tumor's proximity to critical neurovascular structures. Systemic chemotherapy is generally reserved for palliative purposes as it has not shown significant efficacy in ACC​ ([NCBI](https://www.ncbi.nlm.nih.gov/books/NBK557855/))​​ ([IIAR Journals](https://ar.iiarjournals.org/content/37/6/3045))​.   These unique clinical characteristics of ACC necessitate a tailored approach to diagnosis and management, involving a multidisciplinary team to optimize treatment outcomes and manage the complex nature of this malignancy​ ([MDPI](https://www.mdpi.com/2077-0383/13/1/267))​​ ([Springer](https://link.springer.com/article/10.1007/s12105-020-01210-7))​.  For further reading, the sources provide detailed insights:   - StatPearls on NCBI ([www.ncbi.nlm.nih.gov](http://www.ncbi.nlm.nih.gov)) - Anticancer Research (ar.iiarjournals.org) - MDPI's Journal of Clinical Medicine ([www.mdpi.com](http://www.mdpi.com)) - Head and Neck Pathology (link.springer.com) | Seeker: I am a fifth-year resident specializing in head and neck surgery.  Mission: I need your help to prepare for my final residency exam.  AI Role: You are a full professor of head and neck surgery at the most prestigious university in the world.  Register: correct and specialized scientific language. The information must be based on the most recent and solid scientific evidence. I would like you to also provide me with the bibliographical references from which you draw your information  Targeted question: What are the peculiar clinical characteristics of adenoid cystic carcinomas of the salivary glands that differentiate them from other types of tumors at these sites? | Adenoid cystic carcinoma (ACC) of the salivary glands exhibits several clinical characteristics that distinguish it from other salivary gland tumors. Here are the key peculiarities of ACC:   1. **Histopathological Features**:    - ACC is characterized by its distinct histological patterns, notably the cribriform ("Swiss cheese") pattern, which is the most common. Tubular and solid patterns are also observed.    - The presence of perineural invasion is a hallmark feature of ACC, contributing to its high propensity for local recurrence and difficulty in achieving clear surgical margins. 2. **Growth Pattern and Spread**:    - ACC typically exhibits a slow but relentless growth pattern. Despite its slow progression, it has a high tendency for perineural invasion, which leads to significant local destruction and challenges in complete surgical resection.    - Distant metastasis is common, even in the absence of significant local disease progression. The lungs are the most frequent site of distant metastasis, followed by bones and liver. 3. **Clinical Presentation**:    - Patients often present with non-specific symptoms like a painless mass in the salivary gland region. As the disease progresses, symptoms related to perineural invasion, such as pain, numbness, or muscle weakness, may become prominent.    - Facial nerve involvement is common, particularly with tumors originating in the parotid gland, leading to facial palsy. 4. **Prognostic Factors**:    - ACC is notorious for its high rate of late recurrences and distant metastases, which can occur many years after initial treatment. Therefore, long-term follow-up is crucial.    - Tumor stage, histological grade, and the presence of solid histological pattern are important prognostic factors. The solid pattern is associated with a worse prognosis compared to cribriform and tubular patterns. 5. **Treatment and Management**:    - Surgical resection with clear margins is the primary treatment. However, the infiltrative nature and perineural invasion often make complete resection challenging.    - Postoperative radiotherapy is commonly employed to reduce the risk of local recurrence, particularly in cases with positive margins or perineural invasion.    - Chemotherapy has limited efficacy in ACC, and its use is typically reserved for palliative treatment in metastatic disease.   **Bibliographic References:**   1. **Ellington CL, Goodman M, Kono SA, et al.** Adenoid cystic carcinoma of the head and neck: incidence and survival trends based on 1973-2007 surveillance, epidemiology, and end results data. *Cancer*. 2012;118(18):4444-4451. doi:10.1002/cncr.27408. 2. **Jaso J, Malhotra R.** Adenoid cystic carcinoma. *Arch Pathol Lab Med*. 2011;135(4):511-515. doi:10.5858/135.4.511. 3. **Spiro RH.** Distant metastasis in adenoid cystic carcinoma of salivary origin. *Am J Surg*. 1997;174(5):495-498. doi:10.1016/S0002-9610(97)00189-8. 4. **Yarbrough WG, Panaccione A, Chang MT, Ivanov SV.** Clinical and molecular insights into adenoid cystic carcinoma: Neural crest-like stemness as a target. Laryngoscope Investig Otolaryngol. 2016 Aug 4;1(4):60-77. doi: 10.1002/lio2.22.   These references provide detailed insights into the clinical behavior, histopathological features, treatment, and prognosis of adenoid cystic carcinoma of the salivary glands, distinguishing it from other salivary gland tumors. |
|  |  | QAMAI Scores: Reviewer 1: 23; Reviewer 2: 24; Reviewer 3: 25 | | QAMAI Scores: Reviewer 1: 25; Reviewer 2: 26; Reviewer 3: 28 | |
| Theoretical question | 6 | What are the available techniques for bone reconstruction of the mandible after mandibulectomy? Please provide me with the bibliographical references from which you draw your information. | Mandibular reconstruction after mandibulectomy employs a variety of techniques, each tailored to the specific needs of the patient. Here are some of the primary methods:   1. **Free Vascularized Bone Grafts**:    - **Fibula Free Flap**: This is the most common technique, involving the transplantation of the fibula bone along with its blood vessels, which allows for robust structural support and vascularization of the graft.    - **Iliac Crest Free Flap**: Bone from the iliac crest (pelvis) is used, providing a large volume of bone which can also include skin and muscle for more complex reconstructions.    - **Scapula Free Flap**: Bone from the scapula can be harvested along with skin and muscle, useful for reconstructions requiring significant soft tissue. 2. **Non-Vascularized Bone Grafts**:    - **Autogenous Bone Grafts**: Bone harvested from the patient’s own body, such as the iliac crest or rib, used primarily for smaller defects.    - **Allogenic Bone Grafts**: Donor bone from cadavers, which is less commonly used due to higher failure rates compared to vascularized grafts. 3. **Distraction Osteogenesis**:    - This technique involves gradually moving a bone segment using a distraction device to stimulate new bone growth in the gap created, effectively lengthening the mandible. 4. **Titanium Plates and Meshes**:    - **Reconstruction Plates**: These are used to stabilize the mandible and support bone grafts. They are contoured to fit the patient’s anatomy.    - **Titanium Mesh**: Often used with bone grafts to provide additional support and maintain the shape of the reconstructed mandible. 5. **3D Printing and Custom Implants**:    - Advances in 3D printing allow for custom-made implants tailored to the patient’s specific defect, enhancing the precision and integration of the reconstruction. 6. **Tissue Engineering Approaches**:    - **Stem Cell Therapy**: Utilizes mesenchymal stem cells to promote bone regeneration, often combined with scaffolds or injected directly into the defect site.    - **Growth Factors**: Application of bone morphogenetic proteins (BMPs) to stimulate bone growth and healing. 7. **Soft Tissue Reconstruction**:    - **Local Flaps**: Tissue from nearby areas is used to cover the defect and provide a vascular bed for bone grafts.    - **Microvascular Free Flaps**: Soft tissue flaps from distant sites are transplanted with their blood supply, often combined with bone flaps for comprehensive reconstruction. 8. **Hybrid Techniques**:    - Combining methods such as vascularized bone grafts with titanium plates for enhanced stability and integration.   The choice of technique depends on factors like the size and location of the defect, the patient’s overall health, and the need for immediate function and aesthetics​ ([NCBI](https://www.ncbi.nlm.nih.gov/books/NBK563241/))​​ ([BioMed Central](https://journalotohns.biomedcentral.com/articles/10.1186/s40463-023-00639-4))​​ ([SpringerOpen](https://jkamprs.springeropen.com/articles/10.1186/s40902-018-0175-z))​. | Seeker: I am a fourth-year resident specializing in head and neck surgery.  Mission: I need your help to prepare for my final residency exam.  AI Role: You are a full professor of head and neck surgery at the most prestigious university in the world.  Register: correct and specialized scientific language. The information must be based on the most recent and solid scientific evidence. I would like you to also provide me with the bibliographical references from which you draw your information  Targeted question: What are the available techniques for bone reconstruction of the mandible after mandibulectomy? | Reconstruction of the mandible after mandibulectomy is crucial for restoring both function and aesthetics. The available techniques can be broadly categorized into non-vascularized bone grafts and vascularized bone flaps, with a recent emphasis on the latter due to their superior outcomes in terms of vascularity and integration.  **1. Non-vascularized Bone Grafts**   - **Autogenous Bone Grafts:** Harvested from the patient's iliac crest, rib, or fibula. They are primarily used for small defects due to limited vascularity and higher risk of resorption and infection. - **Allogenic Bone Grafts:** Donor bone tissue, which carries risks of immune rejection and disease transmission, and typically shows lower integration compared to autogenous grafts.   **2. Vascularized Bone Flaps**  These flaps provide a robust blood supply, which enhances healing and integration. The most commonly used vascularized bone flaps include:   - **Free Fibula Flap:** The gold standard for mandibular reconstruction. It offers a long segment of bone, dual skin paddles for soft tissue coverage, and can be contoured to match the mandibular defect. It also provides the potential for dental implant placement.   - **Advantages:** Excellent blood supply, good bone length and quality, and ability to harvest with skin paddles.   - **Disadvantages:** Requires microvascular expertise, donor site morbidity, and longer operative time. - **Scapular Free Flap:** Provides both bone and soft tissue components, with less donor site morbidity compared to the fibula.   - **Advantages:** Versatile with a good bone length and contouring capability.   - **Disadvantages:** Requires repositioning during surgery, which can prolong operative time. - **Iliac Crest Free Flap:** Offers a robust bone segment with potential for simultaneous harvest of a skin paddle.   - **Advantages:** Good bone quality and volume, easy to harvest.   - **Disadvantages:** Donor site morbidity, such as gait disturbance and hernia risk. - **Radial Forearm Free Flap:** Primarily used for smaller defects or when soft tissue reconstruction is also needed.   - **Advantages:** Thin and pliable skin paddle, ease of harvest.   - **Disadvantages:** Limited bone length, donor site cosmetic concerns.   **3. Tissue Engineering and Regenerative Approaches**  Recent advancements in tissue engineering have introduced the use of growth factors, scaffolds, and stem cells to enhance bone regeneration. These techniques are still largely experimental but hold promise for future applications.   - **Bone Morphogenetic Proteins (BMPs):** Used to promote osteogenesis in conjunction with bone grafts or scaffolds. - **Scaffolds:** Biodegradable materials that provide a framework for bone growth. Common materials include hydroxyapatite and tricalcium phosphate. - **Stem Cells:** Mesenchymal stem cells (MSCs) can differentiate into osteoblasts and enhance bone regeneration when seeded onto scaffolds or used with growth factors.   **References**   1. Hidalgo, D. A., & Pusic, A. L. (2002). Free-Flap Mandible Reconstruction: A 10-Year Follow-Up Study. *Plastic and Reconstructive Surgery*, 110(2), 438-449. 2. Vaughan E.D. (2009) Functional outcomes of free tissue transfer in head and neck cancer reconstruction. *Oral Oncology,* ;45(4-5):421-430. 3. Wu, V., Helder, M. N., Bravenboer, N., Ten Bruggenkate, C. M., Jin, J., Klein-Nulend, J., & Schulten, E. A. J. M. (2019). Bone Tissue Regeneration in the Oral and Maxillofacial Region: A Review on the Application of Stem Cells and New Strategies to Improve Vascularization. *Stem cells international*, *2019*, 6279721.   These references provide a comprehensive overview of the current state-of-the-art techniques and their outcomes, helping inform decisions on the most suitable approach for mandibular reconstruction. |
|  |  | QAMAI Scores: Reviewer 1: 27; Reviewer 2: 27; Reviewer 3: 25 | | QAMAI Scores: Reviewer 1: 20; Reviewer 2: 21; Reviewer 3: 23 | |
| Theoretical question | 7 | What are the landmarks to find the main trunk of the facial nerve during a parotidectomy procedure? Please provide me with the bibliographical references from which you draw your information. | During a parotidectomy, identifying the main trunk of the facial nerve is crucial to avoid nerve damage and ensure a successful surgery. Here are the key landmarks used to locate the facial nerve trunk:   1. **Tragal Pointer**: The tragal pointer is a projection of cartilage at the anterior part of the ear. The facial nerve trunk is typically located about 1 cm deep and inferior to the tragal pointer. 2. **Tympanomastoid Suture Line**: This is a bony landmark where the tympanic part of the temporal bone meets the mastoid part. The facial nerve trunk is located just inferior to this suture line. 3. **Posterior Belly of the Digastric Muscle**: The facial nerve trunk is found superior and anterior to the posterior belly of the digastric muscle. 4. **Styloid Process**: The facial nerve trunk is located anterior to the styloid process. 5. **Retrograde Dissection of Distal Branches**: In cases where the distal branches of the facial nerve are identified first, they can be traced back retrogradely to the main trunk.   These anatomical landmarks are essential for safely locating the facial nerve during parotidectomy.  **References**   1. **Guntinas-Lichius, O., Gabriel, B., & Klussmann, J. P. (2006). Facial nerve monitoring during parotidectomy: Technique, reliability, and clinical benefit.** Journal of Surgical Oncology, 94(6), 511-516. doi:10.1002/jso.20777 2. **Laureyns, G., & Belden, C. J. (2010). Anatomy of the facial nerve.** Otolaryngologic Clinics of North America, 43(5), 927-942. doi:10.1016/j.otc.2010.05.006 3. **Weniger, F. G., Calhoun, K. H., & Stiernberg, C. M. (1996). Anatomical localization of the facial nerve using the tragal pointer.** The American Journal of Otolaryngology, 17(3), 172-175. doi:10.1016/S0196-0709(96)90054-6   These references provide detailed information on the anatomical landmarks and the importance of preserving the facial nerve during parotidectomy procedures. | Seeker: I am a fourth-year resident specializing in head and neck surgery.  Mission: I need your help to prepare for my final residency exam.  AI Role: You are a full professor of head and neck surgery at the most prestigious university in the world.  Register: correct and specialized scientific language. The information must be based on the most recent and solid scientific evidence. I would like you to also provide me with the bibliographical references from which you draw your information  Targeted question: What are the landmarks to find the main trunk of the facial nerve during a parotidectomy procedure? | Identifying the main trunk of the facial nerve during a parotidectomy is crucial to avoid nerve injury and ensure successful surgical outcomes. The facial nerve trunk emerges from the stylomastoid foramen and can be located using several anatomical landmarks. Here are the key landmarks and techniques based on recent and robust scientific evidence:  **Landmarks**   1. **Tragal Pointer**: This is a commonly used landmark. The tragal pointer is a projection of cartilage located at the junction of the tragus and the root of the helix of the ear. The facial nerve trunk is typically found approximately 1 cm deep and inferior to the tragal pointer. 2. **Tympanomastoid Suture Line**: This is the suture line between the tympanic and mastoid portions of the temporal bone. The facial nerve trunk is situated around 6-8 mm inferior to this line. 3. **Posterior Belly of the Digastric Muscle**: The facial nerve trunk lies just superior and lateral to the posterior belly of the digastric muscle as it attaches to the digastric groove on the mastoid process. 4. **Styloid Process**: Palpation of the styloid process can aid in locating the facial nerve, which lies lateral to this structure. 5. **Mastoid Process**: The nerve trunk is located medial and anterior to the mastoid process. The posterior auricular artery, which lies just anterior to the mastoid tip, can also serve as a guide. 6. **Inferior Margin of the External Auditory Canal**: The nerve trunk is approximately 1 cm inferior and slightly anterior to the inferior margin of the external auditory canal.   **Technique**   - **Retrograde Dissection**: This involves identifying and following the distal branches of the facial nerve proximally to locate the main trunk. This technique can be particularly useful in revision surgeries or when the anatomy is distorted. - **Anterograde Dissection**: This conventional technique starts from the main trunk and proceeds distally. Identifying the main trunk first is facilitated by the landmarks mentioned above.   **Intraoperative Measures**   - **Facial Nerve Monitor**: Utilizing intraoperative facial nerve monitoring can provide real-time feedback and reduce the risk of nerve injury. This is especially useful in challenging cases or when the anatomical landmarks are not clear. - **Magnification**: Using surgical loupes or an operating microscope can enhance visualization of the nerve and surrounding structures.   **References**   1. Afzali, P., & Ward, B. B. (2019). Management of the Neck in Oral Squamous Cell Carcinoma: Background, Classification, and Current Philosophy. *Oral and maxillofacial surgery clinics of North America*, *31*(1), 69–84. 2. Pather, N., & Osman, M. (2006). **Landmarks of the facial nerve: Implications for parotidectomy.** Surgical and Radiologic Anatomy, 28(2), 170-175. 3. Eisele, D. W., & Wang, S. J. (2009). **Complications in head and neck surgery.** Elsevier Health Sciences.   These landmarks and techniques are well-documented and provide a reliable approach to safely identify the facial nerve trunk during a parotidectomy. Always consider patient-specific anatomical variations and intraoperative findings to adjust the approach accordingly. |
|  |  | QAMAI Scores: Reviewer 1: 23; Reviewer 2: 24; Reviewer 3: 22 | | QAMAI Scores: Reviewer 1: 23; Reviewer 2: 24; Reviewer 3: 25 | |
| Theoretical question | 8 | What are the absolute indications for the surgical treatment of an orbital floor fracture? Please provide me with the bibliographical references from which you draw your information. | Surgical treatment for orbital floor fractures, also known as "blowout fractures," is indicated in certain scenarios to prevent long-term complications and restore function. The absolute indications for surgical intervention typically include:   1. **Enophthalmos**: Significant posterior displacement of the eyeball (greater than 2 mm) which can lead to a sunken appearance of the eye. 2. **Diplopia**: Persistent double vision that does not resolve with conservative management and is related to extraocular muscle entrapment. 3. **Muscle Entrapment**: Entrapment of the inferior rectus muscle or other orbital contents within the fracture, leading to restricted eye movement. 4. **Large Orbital Floor Defect**: A defect greater than 50% of the orbital floor area, which can result in enophthalmos or ocular misalignment if left untreated. 5. **Oculocardiac Reflex**: Symptoms such as bradycardia, nausea, or syncope induced by eye movement due to muscle entrapment.   These indications are based on a combination of clinical symptoms, physical examination, and imaging studies, such as CT scans, which help assess the extent of the fracture and its impact on orbital structures.  **References:**   1. **Manson PN, Grivas A, Rosenbaum A, Vannier M.** Studies on enophthalmos: II. The measurement of orbital injuries and their treatment by quantitative computed tomography. Plast Reconstr Surg. 1986. 2. **Burnstine MA.** Clinical recommendations for repair of orbital facial fractures. Curr Opin Ophthalmol. 2003 Oct;14(5):236-40. 3. **Lee HM, Pomerantz JH, Vyas RM, Dodson TB, Yaremchuk MJ.** Predictors of enophthalmos in isolated orbital floor fractures. J Oral Maxillofac Surg. 2009 Nov;67(11):2380-5. 4. **Dutton JJ.** Management of blowout fractures of the orbital floor: Reconstruction with silicone implants. Ophthalmology. 1991. 5. **Jordan DR, Allen LH, White J, Harvey JT, Pashby RC, Esmaeli B.** Intervention planning for orbital floor fractures. Ophthal Plast Reconstr Surg. 1998.   These references provide a comprehensive overview of the criteria for surgical intervention in orbital floor fractures, emphasizing the importance of addressing functional impairments and preventing long-term complications. | Seeker: I am a fourth-year resident specializing in head and neck surgery.  Mission: I need your help to prepare for my final residency exam.  AI Role: You are a full professor of head and neck surgery at the most prestigious university in the world.  Register: correct and specialized scientific language. The information must be based on the most recent and solid scientific evidence. I would like you to also provide me with the bibliographical references from which you draw your information  Targeted question: What are the absolute indications for the surgical treatment of an orbital floor fracture? | Orbital floor fractures, also known as blowout fractures, are commonly encountered in facial trauma. The decision to surgically treat an orbital floor fracture is based on several indications, primarily aimed at preventing long-term functional and cosmetic sequelae. Here are the absolute indications for the surgical treatment of an orbital floor fracture based on the latest evidence:   1. **Diplopia with Positive Forced Duction Test**:    - Persistent diplopia (double vision) that does not improve within 1-2 weeks, especially when associated with positive forced duction test indicating entrapment of extraocular muscles. 2. **Enophthalmos Greater Than 2 mm**:    - Enophthalmos (posterior displacement of the eye) exceeding 2 mm, which is likely to result in significant cosmetic deformity if not addressed surgically. 3. **Large Fracture Size**:    - Fractures involving more than 50% of the orbital floor are more likely to result in significant enophthalmos and require surgical intervention to restore the orbital volume. 4. **Oculocardiac Reflex**:    - Presence of oculocardiac reflex, characterized by bradycardia, nausea, or syncope due to muscle entrapment, necessitates prompt surgical release. 5. **Orbital Entrapment**:    - Entrapment of the inferior rectus or inferior oblique muscles resulting in limited ocular motility and vertical gaze restriction. 6. **Cosmetic Deformity**:    - Significant cosmetic deformity or asymmetry due to the fracture, especially in cases where the patient desires surgical correction for aesthetic reasons. 7. **Persistent Numbness or Paresthesia**:    - Persistent infraorbital nerve hypoesthesia or paresthesia, especially when associated with a large fracture or displaced fragments.   **References:**   1. **Burnstine, M. A. (2002). Clinical recommendations for repair of orbital facial fractures. Current Opinion in Ophthalmology, 13(5), 236-240.** 2. **Jordan, D. R., Allen, L. H., White, J., & Harvey, J. (1998). Surgical intervention for traumatic hypoglobus. Ophthalmology, 105(1), 160-163.** 3. **Smith, B., & Regan, W. F. (1957). Blow-out fracture of the orbit; mechanism and correction of internal orbital fracture. American Journal of Ophthalmology, 44(5), 733-739.** 4. **Girotto, J. A., Gamble, L., Robertson, B., Sisson, G., & Davidson, J. (1998). Blindness following facial fractures: Case report and review of the literature. Plastic and Reconstructive Surgery, 102(6), 1821-1834.**   These references provide a comprehensive overview of the current guidelines and evidence supporting the surgical indications for orbital floor fractures. |
|  |  | QAMAI Scores: Reviewer 1: 29; Reviewer 2: 29; Reviewer 3: 28 | | QAMAI Scores: Reviewer 1: 29; Reviewer 2: 29; Reviewer 3: 30 | |
| Patient inquiry | 1 | I have been diagnosed with squamous cell carcinoma of the tongue, the surgeon told me that it is necessary to have surgery to remove the tumor and the lymph nodes in the neck and to reconstruct the tongue with tissue taken from the forearm. I told the surgeon that I need to think about it but I don't feel like undergoing the operation. What are the potential consequences if I decide not to have surgery? Please give me your references | I'm very sorry to hear about your diagnosis. Squamous cell carcinoma of the tongue is a serious condition that requires careful consideration and prompt treatment. Deciding not to undergo surgery can have significant consequences, and it's essential to understand these fully before making your decision. Here are the potential consequences if you decide not to have surgery:  **Progression of Cancer**   1. **Local Progression**: The tumor may continue to grow locally, leading to more extensive involvement of the tongue and surrounding structures, which can cause significant pain, difficulty swallowing (dysphagia), and problems with speech. 2. **Regional Spread**: Squamous cell carcinoma can spread to nearby lymph nodes in the neck. This can cause swelling and discomfort and may indicate a more advanced stage of cancer. 3. **Distant Metastasis**: Over time, the cancer can spread to distant organs, such as the lungs, liver, or bones, leading to more severe health problems and a significant reduction in life expectancy.   **Quality of Life**   1. **Pain and Discomfort**: As the tumor grows, it can cause increasing pain and discomfort in the mouth and throat. 2. **Functional Impairments**: The ability to speak, eat, and swallow can be severely impacted, affecting nutrition and overall quality of life. 3. **Infection and Ulceration**: The tumor can become ulcerated and infected, leading to bad breath, bleeding, and further pain.   **Prognosis**   1. **Survival Rates**: Early-stage squamous cell carcinoma of the tongue has a relatively good prognosis with appropriate treatment, including surgery. However, the prognosis significantly worsens as the cancer progresses to more advanced stages. 2. **Complications**: Without treatment, the likelihood of serious complications increases, which can lead to life-threatening conditions.   **Psychological Impact**   1. **Mental Health**: Living with untreated cancer can cause significant anxiety, depression, and emotional distress.   **Medical References**   1. **American Cancer Society**: Provides detailed information on the treatment and prognosis of squamous cell carcinoma of the tongue and the importance of early intervention. 2. **National Cancer Institute**: Offers resources on the treatment options and survival rates associated with different stages of tongue cancer. 3. **Mayo Clinic**: Discusses the implications of untreated oral cancers and the benefits of surgical intervention.   Here are some references for further reading:   - American Cancer Society - Tongue Cancer - National Cancer Institute - Treatment of Oral Cavity Cancer - [Mayo Clinic - Oral and Oropharyngeal Cancer](https://www.mayoclinic.org/diseases-conditions/oral-and-oropharyngeal-cancer/symptoms-causes/syc-20351003)   It's crucial to discuss all your concerns and options with your healthcare provider to make an informed decision. Seeking a second opinion might also provide additional perspective and help you feel more confident about the best course of action. | Seeker: I am a patient  Mission: I need medical advice for a problem that has been diagnosed in me.  AI Role: The world's leading expert in head and neck surgery.  Register: clear and understandable language even for a non-expert. The information must be based on the most recent and solid scientific evidence. I would like you to also provide me with the bibliographical references from which you draw your information  Targeted question: I have been diagnosed with squamous cell carcinoma of the tongue, the surgeon told me that it is necessary to have surgery to remove the tumor and the lymph nodes in the neck and to reconstruct the tongue with tissue taken from the forearm. I told the surgeon that I need to think about it but I don't feel like undergoing the operation. What are the potential consequences if I decide not to have surgery? | I understand that receiving a diagnosis of squamous cell carcinoma (SCC) of the tongue is distressing, and deciding on the appropriate course of treatment can be overwhelming. Let me provide you with detailed information to help you make an informed decision.  **Squamous Cell Carcinoma of the Tongue**  Squamous cell carcinoma is a type of cancer that arises from the squamous cells lining the tongue. It is known for its aggressive nature and tendency to spread (metastasize) to nearby tissues and lymph nodes.  **Standard Treatment Approach**  The standard treatment for SCC of the tongue typically involves:   1. **Surgical Removal of the Tumor**: This includes excision of the tumor itself. 2. **Neck Dissection**: Removal of the lymph nodes in the neck to prevent the spread of cancer. 3. **Reconstruction**: Using tissue from the forearm (or other parts of the body) to reconstruct the tongue, which helps preserve speech and swallowing functions.   **Potential Consequences of Not Undergoing Surgery**   1. **Progression of the Cancer**: Without surgery, the tumor is likely to continue growing. This can lead to increased pain, difficulty swallowing, speaking, and breathing, depending on the tumor's location and size. 2. **Spread to Lymph Nodes and Other Organs**: SCC of the tongue can metastasize to the lymph nodes in the neck and distant organs, making the disease more difficult to treat. 3. **Complications**: As the tumor grows, it can cause significant complications such as bleeding, infection, and severe pain, reducing the quality of life. 4. **Reduced Survival Rate**: The prognosis for untreated SCC of the tongue is poor. Surgical intervention, especially when combined with other treatments like radiation or chemotherapy, significantly improves survival rates.   **Evidence and References**  Several studies and reviews support the need for aggressive treatment of tongue SCC to improve outcomes:   - **National Comprehensive Cancer Network (NCCN) Guidelines**: These guidelines recommend surgical resection and neck dissection for resectable oral cavity cancers, including tongue SCC, followed by adjuvant therapy as necessary . - **A Review by Mücke et al. (2016)**: This review highlights the importance of surgery and neck dissection in improving survival rates in patients with oral SCC . - **Clinical Evidence**: A study by Fridman et al. (2018) underscores the critical role of surgery and subsequent therapy in managing tongue SCC, emphasizing that delays or avoidance of treatment lead to worse outcomes .   **Final Thoughts**  While surgery is understandably daunting, the potential benefits significantly outweigh the risks associated with not treating SCC of the tongue. It's essential to discuss your concerns with your healthcare team, who can provide support, additional information, and perhaps alternative options tailored to your situation.  If you have more questions or need further clarification, please don't hesitate to ask. |
|  |  | QAMAI Scores: Reviewer 1: 25; Reviewer 2: 26; Reviewer 3: 28 | | QAMAI Scores: Reviewer 1: 24; Reviewer 2: 22; Reviewer 3: 25 | |
| Patient inquiry | 2 | A month ago, I underwent the removal of a basal cell carcinoma from the skin of my cheek. The stitches were removed 20 days ago. The scar is still very noticeable, but the surgeon told me that's normal. In your opinion, is there any problem? What can I do to help the scar heal better? | It's common for scars to remain noticeable for some time after surgery, especially within the first month. The healing process varies among individuals and can take several months to a year for scars to fully mature and blend in with the surrounding skin. Here are some steps to help improve scar healing:   1. **Follow Post-Operative Instructions**: Adhere strictly to any post-operative care instructions provided by your surgeon. 2. **Keep the Area Clean and Moisturized**: Use gentle, non-irritating products to clean the scar. Keeping the scar moisturized with products like petroleum jelly or silicone gel can help it heal better. 3. **Protect the Scar from Sun Exposure**: Sun exposure can darken scars, making them more noticeable. Use a high-SPF sunscreen or cover the scar with clothing or a bandage when outside. 4. **Avoid Stretching the Scar**: Try to minimize movement that may stretch the scar, as tension can widen it. 5. **Use Scar Treatment Products**: Over-the-counter products such as silicone sheets or gels, and creams containing onion extract (like Mederma) can help improve the appearance of scars. 6. **Massage the Scar**: Gently massaging the scar several times a day can help break down scar tissue and improve circulation to the area. 7. **Consider Professional Treatments**: If the scar remains prominent, treatments such as laser therapy, steroid injections, or microneedling can be considered after consulting with a dermatologist or plastic surgeon.   **References:**   - **American Academy of Dermatology (AAD)**: Provides comprehensive guidelines on wound care and scar management, emphasizing the importance of sun protection and moisturizing. - **National Health Service (NHS)**: Recommends scar massage and the use of silicone products to improve scar appearance. - **Mayo Clinic**: Offers detailed advice on various scar treatment options, including professional treatments for more severe scarring. - **Journal of Clinical and Aesthetic Dermatology**: Contains studies on the effectiveness of various over-the-counter and professional treatments for scar management.   It's important to have a follow-up appointment with your surgeon to ensure that the scar is healing properly and to discuss any concerns you may have. If you notice any signs of infection (redness, swelling, increased pain, or discharge), contact your healthcare provider immediately. | Seeker: I am a patient  Mission: I need medical advice for a problem that has been diagnosed in me.  AI Role: The world's leading expert in head and neck surgery.  Register: clear and understandable language even for a non-expert. The information must be based on the most recent and solid scientific evidence. I would like you to also provide me with the bibliographical references from which you draw your information  Targeted question: A month ago, I underwent the removal of a basal cell carcinoma from the skin of my cheek. The stitches were removed 20 days ago. The scar is still very noticeable, but the surgeon told me that's normal. In your opinion, is there any problem? What can I do to help the scar heal better? | Healing after the removal of a basal cell carcinoma (BCC) on the cheek, especially regarding the scar, can vary from person to person. Generally, what you are experiencing—a noticeable scar a month after surgery—is normal. Here are some key points and recommendations to help improve scar healing:  **Understanding the Healing Process**   1. **Early Phase (First Month)**: After stitches are removed, the initial redness and elevation of the scar are normal. The tissue is still undergoing active healing. 2. **Intermediate Phase (1-3 Months)**: The scar may remain red, firm, and possibly raised. This is part of the natural remodeling process of collagen in the skin. 3. **Late Phase (3-12 Months and beyond)**: Over time, the scar typically flattens, becomes softer, and the redness fades.   **Recommendations for Better Scar Healing**   1. **Sun Protection**: UV radiation can worsen scar appearance. Use a high-SPF (30 or higher) sunscreen daily and wear a wide-brimmed hat when outdoors. 2. **Moisturization and Massage**: Regularly moisturize the scar with a fragrance-free lotion or a product specifically designed for scar care. Gentle massage can help improve scar elasticity and appearance. 3. **Silicone Gel or Sheets**: Silicone-based products are one of the most evidence-supported treatments for scar improvement. Apply silicone gel or wear silicone sheets as directed. 4. **Avoiding Tension**: Minimize tension on the scar by avoiding activities that stretch the skin on your cheek excessively. 5. **Topical Treatments**: Consider using topical treatments that contain ingredients like onion extract (found in some over-the-counter scar gels) or other products recommended by your surgeon. 6. **Consult Your Surgeon**: If the scar appears unusually raised, discolored, or if you have concerns about its appearance, consult your surgeon. They may suggest additional treatments such as steroid injections, laser therapy, or other interventions.   **Monitoring and Follow-Up**  It is important to have regular follow-ups with your healthcare provider to monitor the scar and ensure there are no complications, such as infection or abnormal scar formation (like hypertrophic scars or keloids).  **Scientific Evidence**   - **Sun Protection**: A study by Tandara and Mustoe (2004) highlights the importance of UV protection in minimizing scar pigmentation and improving overall appearance . - **Silicone Gel Sheets**: A review by Gold et al. (2014) provides evidence supporting the use of silicone gel and sheets in improving the texture, color, and height of scars . - **Massage and Moisturization**: Research by Cho et al. (2010) supports the beneficial effects of scar massage and moisturization in scar treatment .   **References**   1. Tandara, A. A., & Mustoe, T. A. (2004). The role of the epidermis in the control of scarring: evidence for mechanism of action for silicone gel. Journal of Plastic, Reconstructive & Aesthetic Surgery, 57(4), 406-411. 2. Gold, M. H., Berman, B., Clementoni, M. T., Gauglitz, G. G., & Nahai, F. (2014). Updated international clinical recommendations on scar management: part 2—algorithms for scar prevention and treatment. Dermatologic Surgery, 40(8), 825-831.   By following these guidelines, you can aid the healing process and improve the appearance of your scar over time. If you have further concerns, it’s always best to discuss them with your surgeon or a dermatologist who can provide personalized advice and treatment options. |
|  |  | QAMAI Scores: Reviewer 1: 22; Reviewer 2: 24; Reviewer 3: 24 | | QAMAI Scores: Reviewer 1: 25; Reviewer 2: 29; Reviewer 3: 28 | |
| Patient inquiry | 3 | I have to undergo a superficial parotidectomy to remove an adenoma. I am a bit worried. What could be the complications?" | Undergoing a superficial parotidectomy to remove an adenoma involves some potential complications, although it is generally considered a safe procedure. Here are some common and rare complications associated with this surgery:   1. **Facial Nerve Injury**: The most significant risk is damage to the facial nerve, which can result in temporary or, rarely, permanent facial paralysis or weakness. This occurs because the facial nerve runs through the parotid gland. Temporary weakness is more common and typically resolves within a few weeks to months​ ([NCBI](https://www.ncbi.nlm.nih.gov/books/NBK557651/))​​ ([Home](https://www.hopkinsmedicine.org/health/treatment-tests-and-therapies/parotidectomy))​. 2. **Frey’s Syndrome**: This condition causes sweating and flushing in the cheek area when eating. It is due to abnormal nerve regeneration and can be managed with treatments such as botulinum toxin injections​ ([Cleveland Clinic](https://my.clevelandclinic.org/health/treatments/24057-parotidectomy-parotid-gland-tumor-surgery))​​ ([BioMed Central](https://journalotohns.biomedcentral.com/articles/10.1186/s40463-020-00472-z))​. 3. **First Bite Syndrome**: Characterized by sharp pain in the parotid region at the first bite of each meal, this is more common in surgeries involving deeper parts of the parotid gland​ ([NCBI](https://www.ncbi.nlm.nih.gov/books/NBK557651/))​​ ([Home](https://www.hopkinsmedicine.org/health/treatment-tests-and-therapies/parotidectomy))​. 4. **Hematoma and Seroma**: These are collections of blood or fluid at the surgery site. Hematomas may require surgical intervention if they are large, while seromas are usually managed with needle aspiration and compression dressings​ ([NCBI](https://www.ncbi.nlm.nih.gov/books/NBK557651/))​​ ([BioMed Central](https://journalotohns.biomedcentral.com/articles/10.1186/s40463-020-00472-z))​. 5. **Infection**: As with any surgical procedure, there is a risk of infection. This can typically be managed with antibiotics​ ([Home](https://www.hopkinsmedicine.org/health/treatment-tests-and-therapies/parotidectomy))​. 6. **Salivary Fistula or Sialocele**: These are rare complications where saliva leaks through the incision or forms a cyst. They usually resolve with conservative treatment such as drainage and pressure dressings​ ([Home](https://www.hopkinsmedicine.org/health/treatment-tests-and-therapies/parotidectomy))​​ ([BioMed Central](https://journalotohns.biomedcentral.com/articles/10.1186/s40463-020-00472-z))​. 7. **Numbness**: Temporary numbness in the ear, face, or jaw is common, often resolving within a few months. Permanent numbness is rare​ ([Cleveland Clinic](https://my.clevelandclinic.org/health/treatments/24057-parotidectomy-parotid-gland-tumor-surgery))​​ ([Home](https://www.hopkinsmedicine.org/health/treatment-tests-and-therapies/parotidectomy))​. 8. **Aesthetic Concerns**: Cosmetic issues, such as visible scarring or contour defects, can occur, but these are generally minimal with proper surgical technique​ ([BioMed Central](https://journalotohns.biomedcentral.com/articles/10.1186/s40463-020-00472-z))​.   While these potential complications might sound concerning, it's important to discuss them with your surgeon, who can provide specific details based on your individual case and the steps taken to minimize these risks. Proper post-operative care and following your surgeon's instructions will also aid in a smooth recovery. | Seeker: I am a patient  Mission: I need medical advice for a problem that has been diagnosed in me.  AI Role: The world's leading expert in head and neck surgery.  Register: clear and understandable language even for a non-expert. The information must be based on the most recent and solid scientific evidence. I would like you to also provide me with the bibliographical references from which you draw your information  Targeted question: I have to undergo a superficial parotidectomy to remove an adenoma. I am a bit worried. What could be the complications? | A superficial parotidectomy is a surgical procedure to remove a tumor from the parotid gland, typically a benign adenoma. While the procedure is generally safe and effective, it's understandable to have concerns about potential complications. Here’s a detailed overview of possible complications based on recent and solid scientific evidence:  **Common Complications**   1. **Facial Nerve Injury**: The most significant risk is damage to the facial nerve, which runs through the parotid gland. This can lead to weakness or paralysis of the facial muscles on the affected side. The risk varies but is minimized by the surgeon's expertise and careful dissection. 2. **Frey's Syndrome**: This is a condition where sweating occurs on the cheek area when eating. It results from misdirected nerve regrowth and can be managed with topical treatments or Botox injections if it occurs. 3. **Salivary Fistula**: A rare complication where saliva leaks through the skin. This usually resolves on its own or with conservative management. 4. **Infection**: As with any surgery, there is a risk of infection. This is usually managed with antibiotics. 5. **Hematoma**: A collection of blood under the skin can occur, which might need to be drained if significant. 6. **Scarring**: While the incision is usually placed in a natural skin crease to minimize cosmetic impact, some scarring is inevitable. The appearance can improve over time and with appropriate wound care.   **Less Common Complications**   1. **Anesthesia Complications**: Any surgery requiring anesthesia carries risks such as allergic reactions, though these are rare. 2. **Recurrence of Tumor**: While adenomas are typically benign, there is a small risk that the tumor could recur, requiring further treatment. 3. **Numbness**: Temporary or permanent numbness in the ear or cheek area due to nerve damage is possible. 4. **Seroma**: Fluid accumulation at the surgery site which may need aspiration.   **Preoperative and Postoperative Care**   - **Preoperative**: Comprehensive imaging (like MRI or CT scans) and possibly a fine-needle aspiration biopsy will help in planning the surgery. Discussing your full medical history with your surgeon is crucial. - **Postoperative**: Follow your surgeon’s instructions regarding wound care, medication, and activity restrictions. Regular follow-up appointments will help monitor healing and address any complications early.   **References**  For more detailed information, the following articles provide comprehensive reviews of the procedure and its outcomes:   1. Ruohoalho, J., Mäkitie, A. A., Aro, K., Atula, T., Haapaniemi, A., Keski-Säntti, H., Takala, A., & Bäck, L. J. (2017). Complications after surgery for benign parotid gland neoplasms: A prospective cohort study. *Head & neck*, *39*(1), 170–176. 2. Quer, M., Vander Poorten, V., Takes, R. P., Silver, C. E., Boedeker, C. C., de Bree, R., Rinaldo, A., Sanabria, A., Shaha, A. R., Pujol, A., Zbären, P., & Ferlito, A. (2017). Surgical options in benign parotid tumors: a proposal for classification. *European archives of oto-rhino-laryngology : official journal of the European Federation of Oto-Rhino-Laryngological Societies (EUFOS) : affiliated with the German Society for Oto-Rhino-Laryngology - Head and Neck Surgery*, *274*(11), 3825–3836. 3. Bron, L. P., & O’Brien, C. J. (2004). Facial nerve function after parotidectomy. Archives of Otolaryngology–Head & Neck Surgery, 130(7), 849-852.   It's natural to feel apprehensive before any surgical procedure. Discussing these concerns with your surgeon can provide additional reassurance and clarity about your specific case. |
|  |  | QAMAI Scores: Reviewer 1: 24; Reviewer 2: 25; Reviewer 3: 24 | | QAMAI Scores: Reviewer 1: 30; Reviewer 2: 30; Reviewer 3: 26 | |
| Patient inquiry | 4 | My 17-year-old daughter has an infected cyst in her neck. She underwent an ultrasound that resulted in a "thyroglossal duct cyst." What is this? What are the possible treatments? | A thyroglossal duct cyst (TGDC) is a fibrous cyst that forms from a persistent thyroglossal duct. This duct is a remnant from the embryonic development of the thyroid gland, which moves from the back of the tongue to its position in the neck. Sometimes, parts of this duct don't close properly, leading to the formation of a cyst. TGDCs are the most common congenital neck cysts and are usually diagnosed in children or young adults.  **Characteristics of a Thyroglossal Duct Cyst:**   - **Location:** Typically found in the midline of the neck, near the hyoid bone. - **Appearance:** Presents as a smooth, round, and firm mass that can move upwards when swallowing or sticking out the tongue. - **Infection:** These cysts can become infected, leading to swelling, pain, and sometimes abscess formation.   **Symptoms of an Infected Thyroglossal Duct Cyst:**   - Redness and tenderness in the neck - Swelling - Fever - Pain, particularly with movement   **Diagnosis:**   - **Ultrasound:** This is commonly used to confirm the presence of the cyst. - **CT or MRI:** In some cases, these imaging techniques might be employed for better visualization.   **Treatment Options:**   1. **Antibiotics:** If the cyst is infected, antibiotics are typically the first line of treatment to manage the infection. 2. **Drainage:** In cases of abscess formation, surgical drainage may be necessary. 3. **Sistrunk Procedure:** This is the definitive treatment and involves surgical removal of the cyst along with the central portion of the hyoid bone and a tract of tissue up to the base of the tongue to prevent recurrence. This procedure is usually recommended once the infection is resolved.   **Follow-up and Prognosis:**   - **Post-Surgery:** Regular follow-up is important to monitor healing and ensure there is no recurrence. - **Prognosis:** With proper surgical treatment, the prognosis is generally excellent. Recurrence rates are low if the Sistrunk procedure is performed correctly.   **References:**   1. [Mayo Clinic - Thyroglossal duct cyst](https://www.mayoclinic.org/diseases-conditions/thyroglossal-duct-cyst/symptoms-causes/syc-20377533) 2. Cleveland Clinic - Thyroglossal duct cyst 3. Johns Hopkins Medicine - Thyroglossal duct cyst   If you have further questions or need more detailed information, feel free to ask! | Seeker: I am a patient  Mission: I need medical advice for a problem that has been diagnosed in me.  AI Role: The world's leading expert in head and neck surgery.  Register: clear and understandable language even for a non-expert. The information must be based on the most recent and solid scientific evidence. I would like you to also provide me with the bibliographical references from which you draw your information  Targeted question: My 17-year-old daughter has an infected cyst in her neck. She underwent an ultrasound that resulted in a "thyroglossal duct cyst." What is this? What are the possible treatments? | A thyroglossal duct cyst is a congenital anomaly that occurs when the thyroglossal duct, a temporary structure formed during embryonic development, fails to disappear completely. This duct connects the thyroid gland to the tongue base. If parts of this duct remain, they can form cysts, which can become infected.  **Symptoms**   - **Swelling**: Typically located in the middle of the neck. - **Pain and Redness**: Especially if infected. - **Difficulty Swallowing or Breathing**: In more severe cases.   **Diagnosis**  The diagnosis of a thyroglossal duct cyst is usually confirmed through imaging studies like an ultrasound, which your daughter has already undergone. Sometimes, additional imaging such as a CT scan or MRI may be necessary.  **Treatment Options**   1. **Antibiotics**: For infected cysts, antibiotics are often prescribed to address the infection. 2. **Surgical Removal**: The definitive treatment for a thyroglossal duct cyst is surgical excision, known as the Sistrunk procedure. This procedure involves removing the cyst along with part of the hyoid bone to reduce the risk of recurrence. 3. **Drainage**: If the cyst is very large or the infection does not resolve with antibiotics alone, drainage may be required before definitive surgery.   **Surgical Procedure: Sistrunk Operation**   - **Preparation**: General anesthesia is used. - **Incision**: A small incision is made in the midline of the neck. - **Excision**: The cyst, tract, and part of the hyoid bone are removed. - **Recovery**: Post-surgical recovery involves a few weeks of healing, with most patients returning to normal activities within a month.   **Follow-Up**  Regular follow-up is essential to monitor for recurrence or complications. It is also crucial to follow the surgeon's post-operative care instructions closely.  **Prognosis**  The prognosis after the Sistrunk procedure is generally excellent, with a low risk of recurrence if the surgery is done properly.  **Bibliographical References**   1. **Narayana Moorthy S, Arcot R. Thyroglossal duct cyst-more than just an embryological remnant. Indian J Surg. 2011 Jan;73(1):28-31. doi: 10.1007/s12262-010-0171-8.** 2. **Patigaroo SA, Dar NH, Jallu AS, Ahmad R. Thyroglossal Duct Cysts: A Clinicosurgical Experience. Indian J Otolaryngol Head Neck Surg. 2017 Mar;69(1):102-107**   If you have any further questions or need more detailed explanations, feel free to ask. |
|  |  | QAMAI Scores: Reviewer 1: 23; Reviewer 2: 23; Reviewer 3: 20 | | QAMAI Scores: Reviewer 1: 24; Reviewer 2: 29; Reviewer 3: 24 | |
| Patient inquiry | 5 | I am a 53-year-old woman, suffering from hypertension and neck pain for about two years, following a road accident. For about two weeks, I have been feeling a sense of tension in my neck, just below the jaw. The sense of tension becomes actual pain just before meals, associated with modest swelling of the area. What is the most likely cause of my problem? | The symptoms you describe, including the tension in the neck below the jaw that turns into pain before meals and the associated modest swelling, could be indicative of a condition related to the salivary glands, such as sialadenitis or a salivary gland stone (sialolithiasis).  Here are some possible causes and explanations:   1. **Sialadenitis**: This is an inflammation of the salivary glands, often caused by bacterial infections. The swelling and pain can become more pronounced before meals due to increased saliva production, which can be obstructed in the inflamed gland. 2. **Sialolithiasis**: Salivary gland stones can block the ducts of the salivary glands, leading to pain and swelling that typically worsens before meals when saliva production increases but cannot be properly excreted. 3. **Salivary Gland Tumors**: Although less common, tumors in the salivary glands can cause swelling and discomfort in the area. 4. **Temporomandibular Joint (TMJ) Disorders**: These disorders can sometimes cause pain in the neck and jaw area, though they are more typically associated with pain in the joint itself and difficulty with jaw movement.   Given your history of hypertension and the trauma from the road accident, it is also essential to rule out any vascular issues or lingering effects from the injury that might be contributing to your symptoms.  **Recommendations:**   1. **Medical Evaluation**: Consult an ENT specialist (otorhinolaryngologist) or your primary care physician for a thorough examination. They may perform imaging studies such as an ultrasound or a CT scan to check for salivary stones or other abnormalities. 2. **Diagnostic Tests**: Blood tests to check for signs of infection or other systemic conditions might also be recommended. 3. **Hydration and Massage**: Increasing fluid intake and gently massaging the swollen area might help if the cause is related to salivary gland obstruction.   **References:**   - Marchal, F., Dulguerov, P., Becker, M., & Lehmann, W. (2001). "Specificity of parotid sialendoscopy." The Annals of Otology, Rhinology, and Laryngology, 110(10), 1-8. - Murr, A. H., & Altman, K. W. (2001). "Management of patients with salivary gland stones." Otolaryngologic Clinics of North America, 32(5), 819-834. - Koch, M., & Zenk, J. (2010). "Algorithms for treatment of salivary gland obstructions." Otolaryngologic Clinics of North America, 42(6), 1173-1192.   Consulting a healthcare provider is crucial for a definitive diagnosis and appropriate treatment. | Seeker: I am a patient  Mission: I need medical advice for a problem that has been diagnosed in me.  AI Role: The world's leading expert in head and neck surgery.  Register: clear and understandable language even for a non-expert. The information must be based on the most recent and solid scientific evidence. I would like you to also provide me with the bibliographical references from which you draw your information  Targeted question: I am a 53-year-old woman, suffering from hypertension and neck pain for about two years, following a road accident. For about two weeks, I have been feeling a sense of tension in my neck, just below the jaw. The sense of tension becomes actual pain just before meals, associated with modest swelling of the area. What is the most likely cause of my problem? | I'm sorry to hear about the discomfort you're experiencing. Based on the symptoms you've described—tension and pain in the neck below the jaw, especially before meals, and associated swelling—one possible cause could be **sialolithiasis**, which is the formation of salivary stones.  **Understanding Sialolithiasis**  Sialolithiasis occurs when calcified stones form within the salivary glands or their ducts. This condition is most common in the submandibular gland, which is located just below the jaw. Here's why this might fit your symptoms:   1. **Pain and Swelling Before Meals**: Salivary stones can block the flow of saliva, causing pain and swelling as the gland tries to produce saliva, especially before and during meals. 2. **Tension and Pain in the Neck**: The blockage can cause a buildup of saliva in the gland, leading to increased pressure and discomfort in the surrounding area. 3. **Swelling**: This can be due to the gland trying to produce saliva, which cannot pass through the duct if it's blocked by a stone.   **Other Possible Causes**  While sialolithiasis is a strong candidate based on your symptoms, other potential causes could include:   - **Sialadenitis**: Infection of the salivary gland, often presenting with pain and swelling. - **Temporomandibular Joint Disorders (TMJ)**: These can cause pain in the jaw and neck area, although they are less likely to cause swelling specifically before meals. - **Lymphadenopathy**: Swelling of lymph nodes due to infection or other conditions, which could be exacerbated by eating.   **Recommended Actions**   1. **Medical Evaluation**: It's crucial to consult with an ENT (ear, nose, and throat) specialist or your primary care physician. They can perform a physical examination and potentially imaging studies, such as an ultrasound or a CT scan, to confirm the presence of a salivary stone. 2. **Hydration and Massage**: Staying well-hydrated and gently massaging the affected area can sometimes help to pass small stones. 3. **Lemon Candy or Citrus**: Sucking on lemon candy or citrus fruits can stimulate saliva flow, which may help to dislodge the stone.   **Treatment Options**  If a stone is confirmed, treatment may involve:   - **Manual Manipulation**: A doctor may be able to massage the stone out. - **Sialogogues**: Medications or substances that increase saliva flow. - **Minimally Invasive Procedures**: Such as sialendoscopy, to remove the stone.   **Bibliographical References**  Here are some scientific references for further reading:   1. **Marchal F, Dulguerov P, Becker M, Barki G, Disant F, Lehmann W.** "Submandibular sialolithiasis: submandibular sialolithiasis: Marchal F, et al. Sialendoscopy: a new diagnostic and therapeutic tool for salivary gland disorders. J Otolaryngol. 2000." [PubMed](https://pubmed.ncbi.nlm.nih.gov/10858663/) 2. **Sohn WJ, Hong SJ, Lee SH, Kim HY, Lee YJ, Kim SY.** "Diagnosis and management of sialolithiasis of the submandibular gland: a systematic review. J Oral Maxillofac Surg. 2020." [PubMed](https://pubmed.ncbi.nlm.nih.gov/32336616/) 3. **Chin SC, Lin YH, Liu SC.** "Sialolithiasis in the submandibular gland: an analysis of 245 cases. Otolaryngol Head Neck Surg. 2020." [PubMed](https://pubmed.ncbi.nlm.nih.gov/32433168/)   I hope this information helps you understand your condition better and guides you towards appropriate treatment. Please follow up with a healthcare professional for a precise diagnosis and tailored treatment plan. |
|  |  | QAMAI Scores: Reviewer 1: 24; Reviewer 2: 22; Reviewer 3: 24 | | QAMAI Scores: Reviewer 1: 30; Reviewer 2: 30; Reviewer 3: 28 | |
| Patient inquiry | 6 | I am a 63-year-old woman. I suffer from hypercholesterolemia and am taking simvastatin, hypertension for which I take losartan, and severe osteoporosis, being treated with alendronic acid. My dentist removed a tooth two months ago. Since then, the area of the procedure has not yet healed, I have pain, and my gum is still open. What is the most likely diagnosis? please give me also your references | The most likely diagnosis for your condition, given your medical history and the symptoms you describe, is **osteonecrosis of the jaw (ONJ)**. This condition is particularly associated with the use of bisphosphonates like alendronic acid, which you are taking for severe osteoporosis.  **Osteonecrosis of the Jaw (ONJ)** is a serious condition where the jawbone starts to die due to lack of blood flow. It is a known complication in patients who take bisphosphonates, particularly following dental procedures like tooth extraction. The symptoms you describe—persistent pain, non-healing extraction site, and an open gum—are consistent with ONJ.  **References**   1. **Ruggiero, S. L., Dodson, T. B., Assael, L. A., Landesberg, R., Marx, R. E., & Mehrotra, B. (2009). American Association of Oral and Maxillofacial Surgeons position paper on bisphosphonate-related osteonecrosis of the jaws—2009 update. Journal of Oral and Maxillofacial Surgery, 67(5), 2-12.** This paper outlines the risks associated with bisphosphonates, particularly in relation to dental health and the development of ONJ. 2. **Allen, M. R., & Burr, D. B. (2009). The pathogenesis of bisphosphonate-related osteonecrosis of the jaw: So many hypotheses, so few data. Journal of Oral and Maxillofacial Surgery, 67(5), 61-70.** This article explores the various mechanisms through which bisphosphonates might lead to ONJ, providing a comprehensive overview of the condition. 3. **Marx, R. E. (2003). Pamidronate (Aredia) and zoledronate (Zometa) induced avascular necrosis of the jaws: A growing epidemic. Journal of Oral and Maxillofacial Surgery, 61(9), 1115-1117.** This early study identified the link between bisphosphonates and jaw necrosis, leading to further research and awareness of the condition. 4. **Colella, G., Campisi, G., Fusco, V., Agrillo, A., & Dimova, C. (2011). Osteonecrosis of the jaw associated with chronic bisphosphonate therapy: An Italian experience. Annals of Internal Medicine, 155(5), 385-386.** This article discusses the clinical presentation and management of ONJ in patients on long-term bisphosphonate therapy.   If you suspect that you have osteonecrosis of the jaw, it is crucial to seek immediate medical attention from your dentist or healthcare provider. Early diagnosis and intervention are key to managing this condition and preventing further complications. | Seeker: I am a patient  Mission: I need medical advice for a problem that has been diagnosed in me.  AI Role: The world's leading expert in head and neck surgery.  Register: clear and understandable language even for a non-expert. The information must be based on the most recent and solid scientific evidence. I would like you to also provide me with the bibliographical references from which you draw your information  Targeted question: I am a 63-year-old woman. I suffer from hypercholesterolemia and am taking simvastatin, hypertension for which I take losartan, and severe osteoporosis, being treated with alendronic acid. My dentist removed a tooth two months ago. Since then, the area of the procedure has not yet healed, I have pain, and my gum is still open. What is the most likely diagnosis? | Given your medical history and current symptoms, the most likely diagnosis is **Medication-Related Osteonecrosis of the Jaw (MRONJ)**. This condition is a known complication in patients taking bisphosphonates, such as alendronic acid, which you are on for severe osteoporosis. MRONJ is characterized by delayed healing, pain, and exposed bone following dental procedures like tooth extraction.  **Key Points:**   1. **Bisphosphonates and Jaw Health**:    - Bisphosphonates, such as alendronic acid, are used to strengthen bones but can impair the jawbone's ability to heal after trauma, such as a tooth extraction.    - These medications inhibit osteoclasts, the cells responsible for bone resorption, leading to decreased bone turnover and potentially compromised healing. 2. **Clinical Presentation**:    - Persistent non-healing of the extraction site.    - Pain in the affected area.    - Exposed bone or an open gum wound. 3. **Risk Factors**:    - Long-term use of bisphosphonates.    - Recent dental surgery or trauma.    - Concurrent use of corticosteroids or other medications affecting bone metabolism.   **Diagnosis and Management:**   1. **Diagnosis**:    - Clinical examination to observe the non-healing extraction site and any exposed bone.    - Imaging studies (e.g., X-rays, CT scans) to assess bone integrity and rule out other conditions. 2. **Management**:    - **Oral Hygiene**: Maintain excellent oral hygiene to reduce infection risk.    - **Antibiotics**: May be prescribed to control infection if present.    - **Antiseptic Mouth Rinses**: Use chlorhexidine or other antiseptic rinses to manage oral flora.    - **Surgical Intervention**: In severe cases, surgical debridement might be necessary to remove necrotic bone.   **Recommendations:**   - **Immediate Action**: Consult with your dentist or an oral surgeon immediately. They will provide a thorough examination and may refer you to a specialist if MRONJ is suspected. - **Continued Monitoring**: Regular dental check-ups and monitoring of the affected area. - **Medication Review**: Discuss with your physician the continued use of alendronic acid, weighing the benefits for osteoporosis against the risk for MRONJ. They may consider alternative treatments if appropriate.   **Bibliography:**   1. **Ruggiero SL, Dodson TB, Assael LA, et al.** "American Association of Oral and Maxillofacial Surgeons position paper on bisphosphonate-related osteonecrosis of the jaws--2009 update." *J Oral Maxillofac Surg.* 2009 May;67(5 Suppl):2-12. 2. **Khan AA, Morrison A, Hanley DA, et al.** "Diagnosis and management of osteonecrosis of the jaw: a systematic review and international consensus." *J Bone Miner Res.* 2015 Jan;30(1):3-23. 3. **Dodson TB.** "The Frequency of Medication-Related Osteonecrosis of the Jaw and Its Associated Risk Factors." *Oral Maxillofac Surg Clin North Am.* 2015 Nov;27(4):509-16.   Please follow up with a healthcare professional as soon as possible to address your symptoms and receive appropriate care. |
|  |  | QAMAI Scores: Reviewer 1: 23; Reviewer 2: 20; Reviewer 3: 20 | | QAMAI Scores: Reviewer 1: 29; Reviewer 2: 30; Reviewer 3: 28 | |
| Patient inquiry | 7 | For about a month I've had a canker sore on my tongue. My doctor prescribed antibiotic therapy, which didn't provide any benefit. For this reason, I was advised to apply a cortisone gel, but the canker sore continues to hurt and doesn't seem to heal. What is the most likely diagnosis and what should I do? please give me also your references | A persistent canker sore (aphthous ulcer) on the tongue that doesn't respond to antibiotic therapy and cortisone gel could indicate several potential underlying issues. Here are a few possibilities and suggested steps:   1. **Recurrent Aphthous Stomatitis (RAS)**: This is the most common cause of recurrent canker sores. It is usually idiopathic, meaning the exact cause is unknown. Factors such as stress, hormonal changes, food allergies, nutritional deficiencies (like B12, folate, or iron), and underlying systemic diseases could contribute. 2. **Nutritional Deficiencies**: Deficiencies in vitamins and minerals, particularly B12, folate, and iron, can lead to persistent canker sores. Blood tests to check for these deficiencies can be helpful. 3. **Autoimmune Diseases**: Conditions like Behçet's disease or celiac disease can cause recurrent aphthous ulcers. These conditions usually present with additional symptoms like genital ulcers, eye inflammation, or gastrointestinal symptoms. 4. **Oral Lichen Planus**: This chronic inflammatory condition can present with painful oral lesions. It often requires a biopsy for definitive diagnosis. 5. **Infections**: While antibiotics typically target bacterial infections, viral infections (like herpes simplex virus) might cause persistent ulcers. 6. **Oral Cancer**: Though less common, persistent non-healing ulcers should be evaluated for malignancy, especially if associated with risk factors like smoking, alcohol use, or HPV infection.   **Next Steps:**   1. **Referral to a Specialist**: Consider seeing an oral medicine specialist or a dermatologist for a more detailed examination and possibly a biopsy. 2. **Blood Tests**: Test for nutritional deficiencies and autoimmune markers. 3. **Biopsy**: If the sore persists despite conservative treatment, a biopsy may be necessary to rule out malignancy or specific diseases like oral lichen planus.   **References:**   1. Scully, C., & Porter, S. (2008). Oral mucosal disease: Recurrent aphthous stomatitis. British Journal of Oral and Maxillofacial Surgery, 46(3), 198-206. 2. Edgar, N. R., Saleh, D., & Miller, R. A. (2017). Recurrent Aphthous Stomatitis: A Review. Journal of Clinical and Aesthetic Dermatology, 10(3), 26-36. 3. Ship, J. A., Chavez, E. M., & Doerr, P. A. (2000). Recurrent aphthous stomatitis. Quintessence International, 31(2), 95-112. 4. Porter, S. R., & Scully, C. (2005). Recurrent aphthous stomatitis: Clinical outcome. Oral Surgery, Oral Medicine, Oral Pathology, Oral Radiology, and Endodontology, 99(4), 411-418.   These references provide a comprehensive overview of recurrent aphthous stomatitis and associated conditions, guiding further diagnostic and therapeutic approaches. | Seeker: I am a patient  Mission: I need medical advice for a problem that has been diagnosed in me.  AI Role: The world's leading expert in head and neck surgery.  Register: clear and understandable language even for a non-expert. The information must be based on the most recent and solid scientific evidence. I would like you to also provide me with the bibliographical references from which you draw your information  Targeted question: For about a month I've had a canker sore on my tongue. My doctor prescribed antibiotic therapy, which didn't provide any benefit. For this reason, I was advised to apply a cortisone gel, but the canker sore continues to hurt and doesn't seem to heal. What is the most likely diagnosis and what should I do?. | A canker sore (aphthous ulcer) that persists for more than a month and does not respond to antibiotic therapy or cortisone gel might suggest a more complex underlying issue than a simple ulcer. Here are some possible diagnoses and recommendations for further steps:  **Possible Diagnoses**   1. **Chronic Traumatic Ulcer**: This can occur if there's constant irritation from dental appliances, rough teeth, or habitual biting. 2. **Oral Lichen Planus**: A chronic inflammatory condition that can cause persistent sores and discomfort. 3. **Herpetiform Ulceration**: A more severe form of aphthous ulcers that can be numerous and painful. 4. **Nutritional Deficiencies**: Deficiencies in vitamins such as B12, folic acid, or iron can contribute to recurrent or persistent ulcers. 5. **Autoimmune Conditions**: Conditions like Behçet's disease or celiac disease can manifest with oral ulcers. 6. **Infection**: Though less common, viral infections like Herpes Simplex Virus (HSV) or fungal infections might need to be ruled out. 7. **Malignancy**: Rarely, persistent non-healing ulcers might indicate an early sign of oral cancer, especially if associated with other risk factors like smoking or alcohol use.   **Recommended Steps**   1. **Comprehensive Oral Examination**: A thorough evaluation by a specialist, such as an oral surgeon or an otolaryngologist (ENT doctor), is essential. They might perform a detailed inspection of your mouth, teeth, and surrounding tissues. 2. **Biopsy**: If the ulcer persists and there's no clear cause, a biopsy might be necessary to rule out malignancy or other underlying conditions. 3. **Blood Tests**: Checking for nutritional deficiencies, autoimmune markers, and infections can provide valuable insights. 4. **Pain Management**: While awaiting a definitive diagnosis, pain management strategies can include:    - Topical anesthetics (e.g., lidocaine)    - Protective pastes or films    - Avoiding spicy, acidic, or rough foods 5. **Review Medications and Medical History**: Some medications can contribute to oral ulcers. A detailed review of your medical history and current medications might reveal clues.   **Bibliographical References**   1. **Scully, C., & Porter, S. (2008). Oral mucosal disease: Recurrent aphthous stomatitis. *British Journal of Oral and Maxillofacial Surgery, 46*(3), 198-206.**    - This article provides an overview of recurrent aphthous stomatitis, its clinical presentation, and treatment options. 2. **Edgar, N. R., Saleh, D., & Miller, R. A. (2017). Recurrent Aphthous Stomatitis: A Review. Journal of Clinical and Aesthetic Dermatology, 10(3), 26-36.**    - This review highlights the role of nutritional deficiencies in the persistence of chronic oral ulcers.   **Conclusion**  Given the persistence of your ulcer and the lack of response to initial treatments, it is crucial to seek a comprehensive evaluation from a specialist. This will help to accurately diagnose the condition and provide appropriate treatment to relieve your symptoms and address the underlying cause. |
|  |  | QAMAI Scores: Reviewer 1: 24; Reviewer 2: 25; Reviewer 3: 28 | | QAMAI Scores: Reviewer 1: 29; Reviewer 2: 30; Reviewer 3: 28 | |
| Patient inquiry | 8 | I have been diagnosed with squamous cell carcinoma of the tongue, the surgeon told me that after resection it is necessary and to reconstruct the tongue with tissue taken from the forearm or from the thigh. I was diagnosed with squamous cell carcinoma of the tongue, the surgeon told me that the tongue needs to be reconstructed with tissue taken from the forearm or thigh. What are the differences between the two flaps in the rehabilitation of swallowing and chewing after surgery? | The reconstruction of the tongue after resection for squamous cell carcinoma often involves using flaps taken from other parts of the body, typically the forearm (radial forearm free flap) or the thigh (anterolateral thigh free flap). Each type of flap has specific characteristics that may influence the rehabilitation of swallowing and chewing. Here’s a comparative analysis of the two:  **Radial Forearm Free Flap (RFFF)**  **Advantages:**   - **Thin and pliable:** The tissue from the forearm is thin and flexible, making it easier to shape and integrate into the oral cavity. - **Good vascularity:** This flap has a reliable blood supply, which helps in healing and reduces the risk of flap failure. - **Sensory recovery:** It has potential for sensory recovery, which can be beneficial for tongue function.   **Disadvantages:**   - **Donor site morbidity:** The forearm donor site can have significant cosmetic and functional implications, including visible scarring and potential for decreased strength and function in the hand. - **Hair growth:** Some patients may experience unwanted hair growth in the reconstructed tongue if not properly managed.   **Anterolateral Thigh Free Flap (ALTFF)**  **Advantages:**   - **Larger tissue volume:** The thigh provides a larger volume of tissue, which can be advantageous for reconstructing extensive defects. - **Less conspicuous donor site:** The donor site on the thigh is less visible and can be easier to conceal, which might be cosmetically preferable for some patients. - **Versatility:** The ALT flap can be harvested with a varying thickness of tissue, allowing for customization based on the defect size and requirements.   **Disadvantages:**   - **Bulkiness:** The tissue from the thigh can be bulkier, which may require additional thinning to fit properly in the oral cavity, potentially complicating the surgery. - **Technical complexity:** Harvesting the ALT flap can be more technically challenging, and it may have a less predictable blood supply compared to the RFFF. - **Limited sensory recovery:** Sensory recovery in the ALT flap is typically less favorable compared to the RFFF.   **Rehabilitation of Swallowing and Chewing**   - **Radial Forearm Free Flap:** Due to its thinness and pliability, the RFFF generally integrates well with the remaining tongue tissue, allowing for more natural movements and potentially better outcomes in terms of articulation, swallowing, and chewing. - **Anterolateral Thigh Free Flap:** While it can provide sufficient bulk for structural support, its thicker nature may require more surgical adjustments. It may not adapt as seamlessly to the dynamic movements required for effective swallowing and chewing.   **Summary**  The choice between the RFFF and ALTFF depends on the size of the defect, the need for bulk versus pliability, and the patient’s overall condition and preferences. The RFFF is often preferred for its thinness and pliability, which can result in better functional outcomes for speech and swallowing, while the ALTFF is chosen for larger defects or when less visible donor site scarring is a priority.  For detailed, personalized advice, discussing these options with your surgical team, including a head and neck surgeon and a reconstructive surgeon, is crucial. They can consider your specific situation, including the size of the defect, your anatomy, and your rehabilitation goals.  **References**   1. "Radial Forearm Free Flap Versus Anterolateral Thigh Free Flap for Tongue Reconstruction: A Systematic Review and Meta-analysis." Journal of Reconstructive Microsurgery. 2. "Anterolateral Thigh Flap in Head and Neck Reconstruction." Oral Oncology. 3. "Functional Outcomes After Free Flap Reconstruction for Tongue Cancer." [Head & Neck](https://onlinelibrary.wiley.com/doi/10.1002/hed.25073).   These references provide a comprehensive overview of the outcomes and considerations associated with each flap type in the context of tongue reconstruction. | Seeker: I am a patient  Mission: I need medical advice for a problem that has been diagnosed in me.  AI Role: The world's leading expert in head and neck surgery.  Register: clear and understandable language even for a non-expert. The information must be based on the most recent and solid scientific evidence. I would like you to also provide me with the bibliographical references from which you draw your information Targeted question: I have been diagnosed with squamous cell carcinoma of the tongue, the surgeon told me that after resection it is necessary and to reconstruct the tongue with tissue taken from the forearm or from the thigh. I was diagnosed with squamous cell carcinoma of the tongue, the surgeon told me that the tongue needs to be reconstructed with tissue taken from the forearm or thigh. What are the differences between the two flaps in the rehabilitation of swallowing and chewing after surgery? | Squamous cell carcinoma (SCC) of the tongue is a common type of oral cancer, often requiring surgical resection to remove the tumor. Post-surgical reconstruction is critical to restore function, particularly for swallowing and chewing. Two commonly used methods for reconstruction are the radial forearm free flap (RFFF) and the anterolateral thigh (ALT) flap. Here, I'll explain the differences between these two options and their implications for rehabilitation.  **Radial Forearm Free Flap (RFFF)**  **Advantages:**   - **Tissue Thinness and Flexibility:** The radial forearm provides thin, pliable skin that can be molded effectively to reconstruct intricate structures of the tongue, which is beneficial for speech and swallowing functions. - **Vascularity:** It has a reliable vascular supply, which ensures good blood flow to the reconstructed tissue, promoting healing and integration. - **Donor Site Morbidity:** The donor site is generally well tolerated, though it may require a skin graft to close.   **Disadvantages:**   - **Functional Deficit at Donor Site:** There can be some functional impairment and cosmetic concerns at the forearm donor site, including reduced wrist movement or noticeable scarring. - **Limited Bulk:** The radial forearm flap may not provide sufficient bulk for larger defects, which could be a limitation for extensive reconstructions.   **Anterolateral Thigh (ALT) Flap**  **Advantages:**   - **Versatility in Tissue Thickness:** The ALT flap can be harvested with varying thickness, making it suitable for different defect sizes and needs. - **Bulkier Tissue:** It can provide more bulk than the RFFF, which is beneficial for filling larger defects and maintaining contour and function. - **Minimal Donor Site Morbidity:** The donor site morbidity is low, with less noticeable scarring and minimal impact on thigh function.   **Disadvantages:**   - **Technical Complexity:** The ALT flap can be technically more challenging to harvest due to anatomical variations in the perforator vessels. - **Thicker Tissue:** The tissue from the thigh is generally thicker and may not be as easily molded as the forearm tissue, which can affect the precision of tongue reconstruction and subsequently, the functional outcomes for speech and swallowing.   **Rehabilitation of Swallowing and Chewing**  **Swallowing:**   - **RFFF:** Due to its thin and pliable nature, the RFFF typically allows for better restoration of tongue mobility, which is crucial for effective swallowing. This can lead to quicker and more effective rehabilitation. - **ALT Flap:** While the ALT flap provides more bulk, it may be less adaptable for precise movements, potentially making the initial phase of swallowing rehabilitation more challenging. However, with targeted therapy, patients can achieve satisfactory outcomes.   **Chewing:**   - **RFFF:** The thin nature of the flap can make chewing easier to adapt to, especially for fine motor movements required for manipulating food. - **ALT Flap:** The additional bulk from the ALT flap can aid in reconstructing larger defects and maintaining tongue strength, which is beneficial for chewing harder foods. However, the initial adaptation period may be longer.   **Conclusion**  The choice between the radial forearm free flap and the anterolateral thigh flap largely depends on the size and location of the defect, patient-specific factors, and the surgeon's expertise. Both flaps have their unique advantages and challenges in terms of rehabilitation for swallowing and chewing. A thorough discussion with your surgical team, including a head and neck surgeon and a speech therapist, can help tailor the best approach for your specific situation.  **Bibliographical References**   1. **Liu, X., et al.** (2016). "Reconstruction of tongue defects: Comparison of functional outcomes with radial forearm versus anterolateral thigh free flap." *J Oral Maxillofac Surg*, 74(10), 1940-1947. 2. **Vincent, A., Kohlert, S., Lee, T. S., Inman, J., & Ducic, Y. (2019).** Free-Flap Reconstruction of the Tongue. *Seminars in plastic surgery*, *33*(1), 38–45. 3. **Huang, C. H., Chen, H. C., Huang, Y. L., Mardini, S., & Feng, G. M. (2004).** Comparison of the radial forearm flap and the thinned anterolateral thigh cutaneous flap for reconstruction of tongue defects: an evaluation of donor-site morbidity. *Plastic and reconstructive surgery*, *114*(7), 1704–1710.   These references provide a comprehensive comparison of functional outcomes for tongue reconstruction using the RFFF and ALT flap, based on recent and robust scientific evidence. |
|  |  | QAMAI Scores: Reviewer 1: 25; Reviewer 2: 26; Reviewer 3: 24 | | QAMAI Scores: Reviewer 1: 25; Reviewer 2: 25; Reviewer 3: 25 | |
